# How Do Self-Interaction Errors Associated with Stretched Bonds Affect Barrier Height Predictions?

**DOI:** 10.1021/acs.jpca.2c07894

**Published:** 2023-02-14

**Authors:** Priyanka B. Shukla, Prakash Mishra, Tunna Baruah, Rajendra R. Zope, Koblar A. Jackson, J. Karl Johnson

**Affiliations:** † Department of Chemical & Petroleum Engineering, University of Pittsburgh, Pittsburgh, Pennsylvania 15261, United States; ‡ Computational Science Program, University of Texas at El Paso, El Paso, Texas 79968, United States; § Department of Physics, University of Texas at El Paso, El Paso, Texas 79968, United States; ∥ Physics Department and Science of Advanced Materials Program, Central Michigan University, Mount Pleasant, Michigan 48859, United States

## Abstract

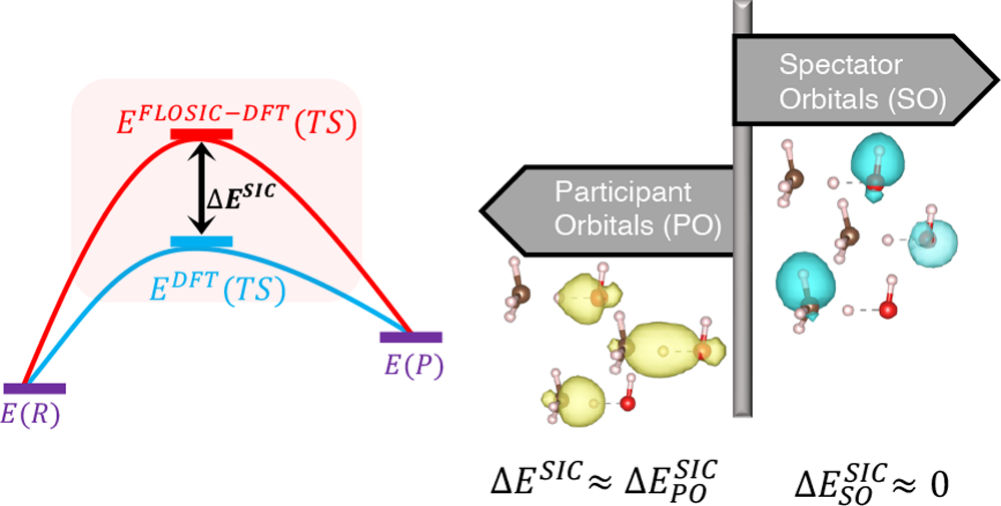

Density functional theory (DFT) suffers from self-interaction errors (SIEs) that generally result in the underestimation of chemical reaction barrier heights. This is commonly attributed to the tendency of density functional approximations to overstabilize delocalized densities that typically occur in the stretched bonds of transition state structures. The Perdew–Zunger self-interaction correction (PZSIC) and locally scaled self-interaction correction (LSIC) improve the prediction of barrier heights of chemical reactions, with LSIC giving better accuracy than PZSIC on average. These methods employ an orbital-by-orbital correction scheme to remove the one-electron SIE. In the context of barrier heights, this allows an analysis of how the self-interaction correction (SIC) for each orbital contributes to the calculated barriers using Fermi–Löwdin orbitals (FLOs). We hypothesize that the SIC contribution to the reaction barrier comes mainly from a limited number of orbitals that are directly involved in bond-breaking and bond-making in the reaction transition state. We call these participant orbitals (POs), in contrast to spectator orbitals (SOs) which are not directly involved in changes to the bonding. Our hypothesis is that Δ*E*_Total_^SIC^ ≈ Δ*E*_PO_^SIC^, where Δ*E*_Total_^SIC^ is the difference in the SIC corrections for the reactants or products and the transition state. We test this hypothesis for the reaction barriers of the BH76 benchmark set of reactions. We find that the stretched-bond orbitals indeed make the largest individual SIC contributions to the barriers. These contributions increase the barrier heights relative to LSDA, which underpredicts the barrier. However, the full stretched-bond hypothesis does not hold in all cases for either PZSIC or LSIC. There are many cases where the total SIC contribution from the SOs is significant and cannot be ignored. The size of the SIC contribution to the barrier height is a key indicator. A large SIC correction is correlated to a large LSDA error in the barrier, showing that PZSIC properly gives larger corrections when corrections are needed most. A comparison of the performance of PZSIC and LSIC shows that the two methods have similar accuracy for reactions with large LSDA errors, but LSIC is clearly better for reactions with small errors. We trace this to an improved description of reaction energies in LSIC.

## 1 Introduction

Density functional approximations (DFAs) suffer from self-interaction errors (SIEs) due to the incomplete cancellation of the self-Coulomb and self-exchange-correlation (XC) energies for one-electron densities. This leads to a failure to accurately predict properties such as electron removal energies, molecular dissociation energies, and others.^1−7^ SIEs particularly affect the total energy of systems with stretched bonds such as the transition states (TS) of chemical reactions.^3,4,8,9^ Standard DFAs predict an unphysical lowering of the total energy for delocalized charge distributions.^3,10^ In transition states, orbitals corresponding to stretched or breaking bonds can be spread over multiple atoms. For example, in the model reaction AB + C → A + BC, orbitals in the transition state may spread over all three atoms, A, B, and C, whereas the orbitals are confined to AB or C in the reactants or A or BC in the products. Because of the delocalized nature of the density related to the stretched bonds, DFT underpredicts the energy of the transition state relative to the reactants or the products, making the forward and reverse reaction barriers too small.

The Perdew–Zunger self-interaction correction (PZSIC)^11^ has been found to give improved predictions for reaction barrier heights.^3,12^ PZSIC removes electron self-interaction on an orbital-by-orbital basis and is exact in the limit of a one-electron density. The related locally scaled self-interaction correction (LSIC) method of Zope et al.^13^ is also an orbital-by-orbital correction scheme. It restores an exact description of the uniform electron gas, a key feature of standard DFAs that is lost in PZSIC,^14^ while maintaining the exact treatment of one-electron systems. By doing so, LSIC provides improved predictions of properties such as electron affinities, ionization energies, atomization energies, and polarizabilities^15−18^ compared to the uncorrected DFA, whereas PZSIC can overcorrect. Recently, Mishra et al.^19^ studied the performance of PZSIC and LSIC (with two different scaling schemes LSIC(*z*) and LSIC(*w*)^20,21^) for barrier heights of chemical reactions using the large BH76 benchmark set originally developed by Truhlar and co-workers^22,23^ and updated in a recent database.^24^ BH76 contains reference values of forward and reverse barriers for 19 hydrogen transfer (HT) reactions and 19 non-hydrogen transfer (NHT) reactions. Mishra and co-workers found that both PZSIC and LSIC significantly improve the prediction of barrier heights compared to the uncorrected local spin density approximation (LSDA) results, with LSIC performing better overall.

Because PZSIC and LSIC are orbital-by-orbital correction schemes, the contributions of individual orbitals to the overall correction of chemical barrier heights can be determined. The Fermi–Löwdin orbitals (FLOs) used in the FLOSIC implementation of the self-interaction correction^25^ are the proper orbitals to use for this purpose. The FLOs are localized orbitals used to define the SIC terms in the PZSIC total energy expression (vide infra) and are optimized to minimize the PZSIC energy. As noted above, PZSIC is exact for any one-electron density and is in that sense free of one-electron self-interaction. For a many-electron system, it can be thought of as removing the one-electron SIE (although many-electron SIE can still remain^26^). Examining the corrections due to individual FLOs thus gives insight into the sources of the one-electron SIE affecting barrier heights.

We hypothesize that the total barrier correction is dominated by contributions from the stretched-bond orbitals in the transition states. We refer to these below as participant orbitals (POs), in contrast to the spectator orbitals (SOs) that are not involved in bonding changes. We further expect that the stretched-bond hypothesis will be satisfied most consistently when the SIC contribution to the barrier is large. Barriers for which the SIC correction is small are cases where self-interaction effects are small and the effects of stretched-bonds are not expected to be as important.

The focus of this paper is to test the stretched-bond hypothesis using the results obtained by Mishra et al.^19^ for BH76. The analysis consists of identifying the POs and SOs in the transition state and the corresponding orbitals in the reactant or product states and comparing the SIC energies of these orbitals to determine their contributions to the overall reaction barrier correction. Our results show that the most delocalized PO in the transition state is the largest source of SIE for a given reaction. The correction associated with that PO is positive, increasing the height of the barrier, which is typically predicted to be too small by LSDA. The stretched-bond hypothesis holds in most cases where the overall SIC barrier corrections are large, but it does not always hold. The total contribution of the SOs to the barrier height can be significant and generally cannot be overlooked, even though the per-orbital corrections for the POs are typically much larger. The findings are similar for LSIC, but the hypothesis holds in a smaller fraction of cases. Our analysis also shows that LSIC predicts barriers more accurately than PZSIC where the overall barrier correction is small and that this accounts for the overall better performance of LSIC for the BH76 set.

## 2 Theory and Methods

The PZSIC^11^ total energy is given by
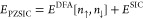
1
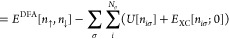
2where *E*^DFA^[*n*_*↑*_, *n*_*↓*_] is the conventional DFA total energy that depends only on *n*_*↑*_, and *n*_*↓*_, the total electron densities for the up and down spin channels. *E*^SIC^ is the SIC correction energy, which consists of subtracting the self-Coulomb *U*[*n*_*i*σ_] and self-exchange correlation *E*_XC_[*n*_*i*σ_; 0]) energies for the *N*_σ_ orbitals of each spin. *n*_*i*σ_ is the density of the *i*th orbital of spin σ. We use the Fermi–Löwdin orbital self-interaction correction (FLOSIC) scheme^25,27−29^ to obtain orbitals that minimize the PZSIC total energy. The Fermi–Löwdin orbitals (FLOs) are localized and reflect the chemistry of the molecules. For example, single- and multiple-bond FLOs can be identified, as can lone pair and radical orbital FLOs, etc. The orthonormal FLOs are created via a unitary transformation on the canonical Kohn–Sham orbitals. The transformation depends on parameters known as Fermi orbital descriptors (FODs). In a FLOSIC calculation, the FODs are optimized to produce FLOs corresponding to the minimum FLOSIC total energy. More details on obtaining FLOs can be found in previous publications^27,30^ and in the work of Mishra et al.^19^

In the LSIC method of Zope et al.,^13^ the self-Coulomb and self-exchange correlation energies in eq 2 are replaced by the locally scaled LSIC counterparts
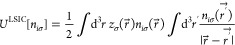
3and
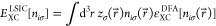
4where ε_XC_^DFA^ is the DFA exchange energy density and  is a scaling factor chosen to range between zero, for a uniform density, and one, for a one-electron density. (To obtain the corresponding expressions for PZSIC, simply set  to one in the preceding equations.) In practice, the scaling reduces the correction in regions where the density is slowly varying but applies it at full strength where the density is one-electron-like. *E*^LSIC^ is evaluated on the self-consistent PZSIC density. Recent studies show^31^ that using a self-consistent LSIC density does not change LSIC results significantly.

The PZSIC/LSIC energies *E*(X) for chemical reaction barriers, where X = reactants (R), products (P), and transition states (TS), can then be written as
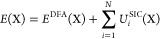
5where  is the total SIC energy of the *i*th FLO in X. The spin labels are omitted here for simplicity. Forward (F) and reverse (R) reaction barriers are
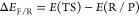
6
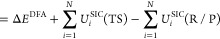
7
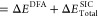
8The SIC contribution to the reaction barrier heights Δ*E*_Total_^SIC^ can be further analyzed by considering contributions from the POs and SOs,
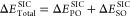
9
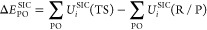
10
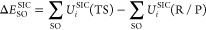
11The precision of the Δ*E*^SIC^ calculation is about 1 × 10^–4^ hartree, so we set any number having this value or smaller to zero in all the tables.

We tested the stretched bond hypothesis using the results of Mishra et al.^19^ for the BH76 reaction barrier benchmark set. These were obtained using the FLOSIC code,^32^ which is based on the NRLMOL code.^33,34^ All FLOSIC calculations studied here are spin-unrestricted except those for CH_4_, H_2_O, H_2_, and HF molecules. All references to PZSIC energies in the remainder of the paper are to PZSIC–LSDA results. We excluded two of the BH76 reactions from our analysis because they have negative barriers, an artifact of using isolated reactants or products when one or more species is charged. Also, we included the barriers of the forward direction only for the eight symmetric reactions in BH76, in order to have a unique set of barriers. In total, we studied 66 distinct reaction barriers taken from the BH76 data set. Thirty of the 66 are NHT reactions (with individual reactions denoted as TN*n*, where *n* is the reaction number), and the remainder are HT reactions (denoted as T*n*). To check whether the hypothesis is more likely to hold when Δ*E*_Total_^SIC^ is large, we divided the reactions into two sets: (1) Δ*E*_Total_^SIC^ > 10 kcal/mol and (2) Δ*E*_Total_^SIC^ < 10 kcal/mol based on the PZSIC–LSDA energy. HT and NHT reactions appear in both sets. We chose the threshold of 10 kcal/mol because the average PZSIC energy correction for all the reactions studied is 10.14 kcal/mol and because there is a gap in the distribution of Δ*E*_Total_^SIC^ values at around 10 kcal/mol. Thirty-three of the 66 reaction barriers have large PZSIC corrections, i.e., Δ*E*_Total_^SIC^ > 10 kcal/mol.

To evaluate our hypothesis, we adopt the criterion that 1.33 ≥ Δ*E*_PO_^SIC^/Δ*E*_Total_^SIC^ ≥ 0.67. This is equivalent to the statement that Δ*E*_PO_^SIC^ ≥ 2Δ*E*_SO_^SIC^ when both are positive and  when Δ*E*_SO_^SIC^ < 0.

## 3 Results and Discussion

In this section, we present the results of our study of the stretched bond hypothesis. We illustrate the analysis by showing the details for one example of a reaction where the hypothesis is satisfied and one where it is not. We also present results comparing the accuracy of PZSIC and LSIC barrier height predictions when Δ*E*_Total_^SIC^ is large and small.

### 3.1 Stretched Bond Hypothesis Test

Testing the hypothesis for a reaction involves first identifying the bonds that are being broken or formed in the TS. The FLOs associated with these active bonds are then indentified by visual inspection of FLO density plots and are designated the POs, while the rest are the SOs. In Tables S1 and S2, we list the active bonds in all of the NHT and HT reactions, respectively. The corresponding POs and SOs of the R and P are then determined on a one-to-one basis by identifying the FLOs that evolve into the POs and SOs of the TS. While not automated, this mapping is straightforward given a knowledge of the bonding and the clear connection of the localized FLOs to bonding orbitals, lone pair orbitals, etc. The number of POs depends on the reaction.

**Figure 1:**
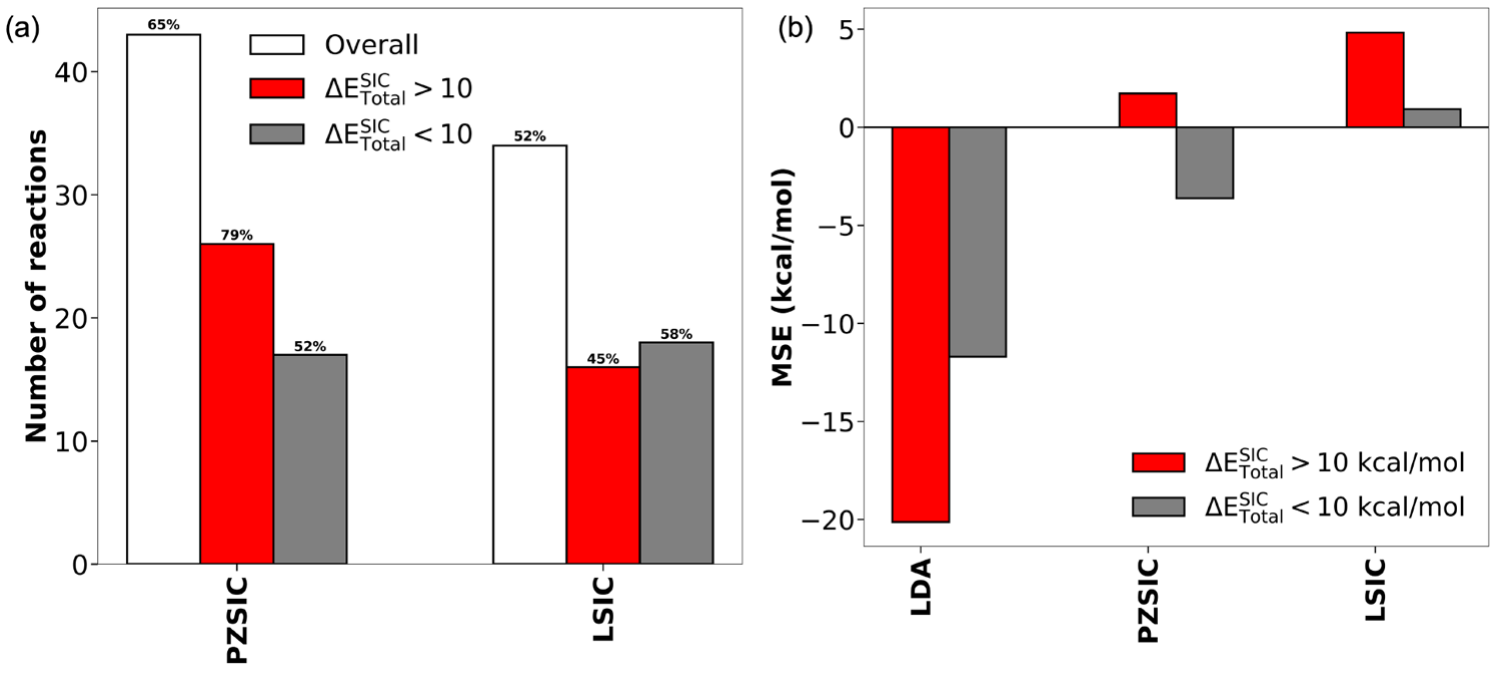
(a) Number of reactions that follow the stretched-bond hypothesis for PZSIC and LSIC. Results for the overall set of 66 reactions are shown, along with the 33 reactions for which the PZSIC correction to the barrier height is large and the 33 reactions where the correction is small, Δ*E*_Total_^SIC^ > 10 and < 10 kcal/mol, respectively. (b) Mean signed error (MSE) for LSDA, PZSIC, and LSIC barrier height energies relative to accurate reference values for reactions with large and small corrections.

After the POs and SOs are identified, Δ*E*_PO_^SIC^ and Δ*E*_SO_^SIC^ are computed and used to determine whether the hypothesis is satisfied. In Figure 1a, the number of reactions for which the hypothesis is followed is shown for PZSIC and LSIC. For PZSIC, we find that the hypothesis is satisfied in 79% of the reactions where Δ*E*_Total_^SIC^ > 10 kcal/mol, but in only 52% where Δ*E*_Total_^SIC^ < 10 kcal/mol. Overall, the hypothesis is satisfied in 65% of the reactions in PZSIC. For LSIC (Figure 1a), the hypothesis is followed in 45% of reactions where the barrier correction is large and in 58% when the correction is small. The hypothesis is satisfied in 52% of all reactions in LSIC. The fractional contributions from the POs for all cases where Δ*E*_Total_^SIC^ > 10 and < 10 kcal/mol are given in Tables S3 and S4, respectively. The average fractional contribution of the POs is 0.97 when Δ*E*_Total_^SIC^ > 10 kcal/mol and 1.67 when Δ*E*_Total_^SIC^ < 10 kcal/mol. For LSIC, the corresponding values are 0.88 and 0.84, respectively. The reactions where the hypothesis is/is not followed are listed in Tables S5 and S6, respectively, for PZSIC calculations and in Tables S8 and S9, respectively, for LSIC.

### 3.2 Example 1: A Reaction That Follows the Hypothesis

In this section, we illustrate the analysis of the reaction T4(F) OH + CH_4_ → H_2_O + CH_3_, for which the stretched bond hypothesis is satisfied. The PZSIC contribution to the reaction barrier for the for T4 reaction is Δ*E*_Total_^SIC^ = 17.19 kcal/mol, placing T4(F) in the group of reactions with large barrier corrections.

**Figure 2:**
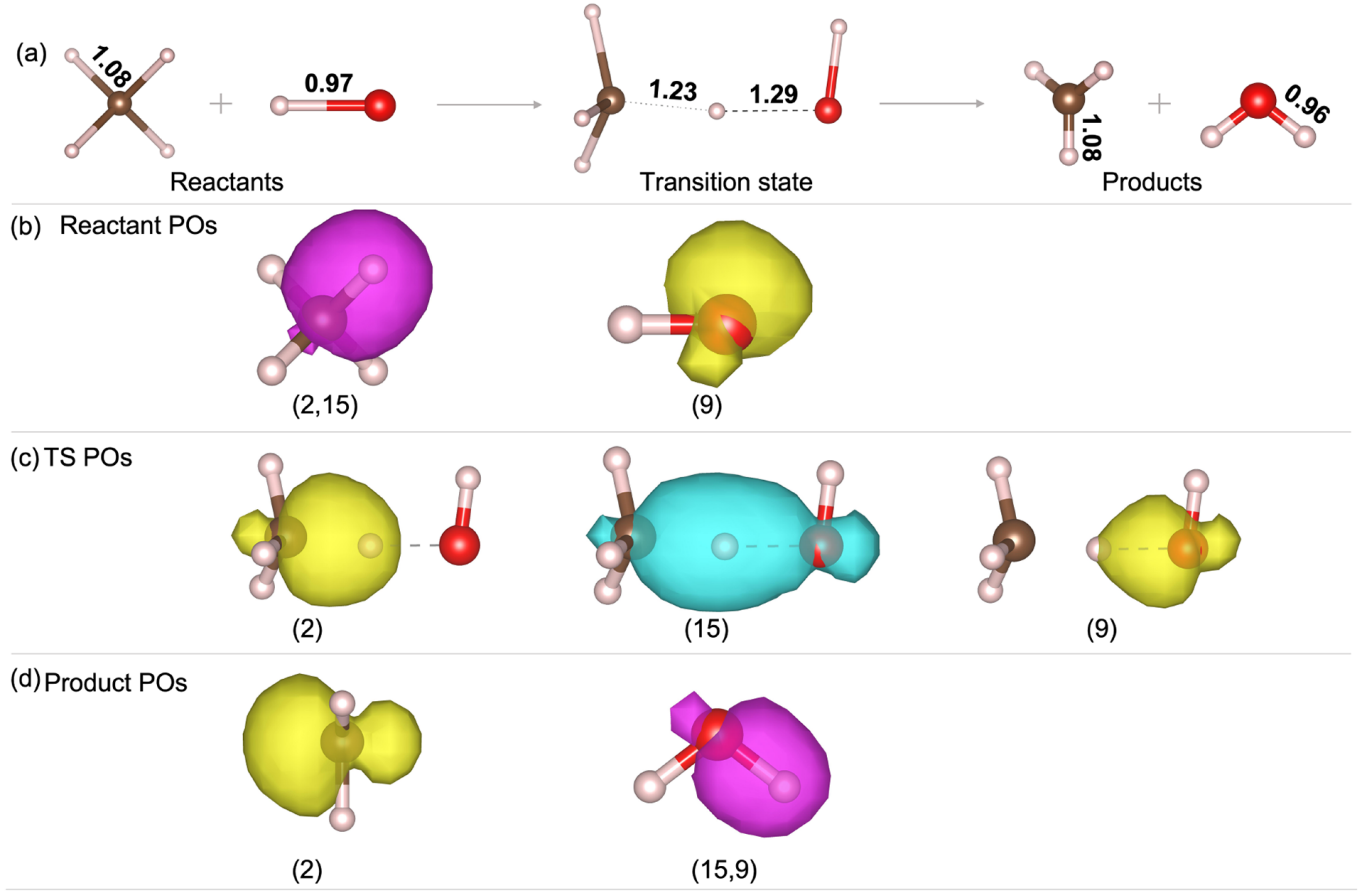
(a) Reaction scheme of T4, OH + CH_4_ → H_2_O + CH_3_. Bond lengths of active bonds are shown in Å. (b–d) Isosurface plots for PO densities in the reactants (b), transition state (c), and products (d). All isosurface values are 0.001/bohr^2^. Color: C (brown), H (pink), O (red), unpolarized POs (magenta), up-spin POs (yellow), and down-spin POs (cyan). The PO labels are the same as in Table 1. The orbital indices shown here were automatically generated by the FLOSIC code for the TS and manually mapped to the corresponding orbitals for R and P. A pair of indices, e.g., (2, 15), denotes a pair of spin-up and spin-down orbitals.

In T4(F), a C–H bond in the reactant molecule CH_4_ breaks, and the freed H atom forms a bond with the O–H radical, producing H_2_O and a CH_3_ radical in the product state. The three POs for this reaction are the 2 FLOs of the breaking C–H bond and the unpaired FLO of the O–H radical. Isosurface plots for the POs of the R, TS, and P are shown in Figure 2. Plots for the SOs are shown in Figure S1 of the Supporting Information. We note that the FLOSIC calculations for each isolated molecule in the reactants or products are performed separately. Many of these reactant or product molecules are symmetric and have symmetry-equivalent orbitals. When one of these orbitals is mapped to a PO in the TS, the choice of which orbital to designate as a PO is arbitrary because each of the symmetry-equivalent orbitals has essentially the same SIC energy. For example, in Figure 2 we could have identified the orbital associated with any of the four C–H bonds as the PO.

The comparison of the SIC energies of the POs and SOs between the reactants and the transition state is shown in Table 1 (the labels shown correspond to those used in Figures 2 and S1). The POs contribute a total Δ*E*_PO_^SIC^(TS–R) of 0.0219 hartree (13.74 kcal/mol) to the forward barrier height, while the SO contribution Δ*E*_SO_^SIC^(TS–R) is only 0.0055 hartree (3.45 kcal/mol). This corresponds to fractional contributions of 0.8 and 0.2 for the POs and SOs, respectively.

**Table 1:** (i) SIC Energies (hartree) Computed in Both PZSIC (*U*_*i*_^SIC^) and LSIC (*U*_*i*_^LSIC^) for the Participant Orbitals (POs) of the Reactants (R) and Transition State (TS) for the Reaction T4(F) OH + CH_4_ → H_2_O + CH_3_^a^

(i) PO SIC Energies						
PO	*U*_*i*_^SIC^(R)	*U*_*i*_^SIC^(TS)	Δ*U*_*i*_^SIC^(TS–R)	*U*_*i*_^LSIC^(R)	*U*_*i*_^LSIC^(TS)	Δ*U*_*i*_^LSIC^(TS–R)
9	–0.0268	–0.0262	0.0006	–0.0250	–0.0195	0.0055
15	–0.0221	–0.0037	0.0184	–0.0176	–0.0053	0.0123
2	–0.0221	–0.0192	0.0029	–0.0176	–0.0132	0.0044
Δ*E*_PO_^SIC^(TS–R)			0.0219			0.0222

(ii) SO SIC Energies						
SO	*U*_*i*_^SIC^(R)	*U*_*i*_^SIC^(TS)	Δ*U*_*i*_^SIC^(TS–R)	*U*_*i*_^LSIC^(R)	*U*_*i*_^LSIC^(TS)	Δ*U*_*i*_^LSIC^(TS–R)
6	–0.2945	–0.2943	0.0002	–0.1945	–0.1962	–0.0017
12	–0.2951	–0.2943	0.0008	–0.2157	–0.2118	0.0039
1	–0.2073	–0.2075	–0.0002	–0.1544	–0.1518	0.0026
11	–0.2073	–0.2073	0.0000	–0.1544	–0.1585	–0.0041
3	–0.0221	–0.0221	0.0000	–0.0176	–0.0174	0.0002
4	–0.0221	–0.0221	0.0000	–0.0176	–0.0174	0.0002
5	–0.0221	–0.0221	0.0000	–0.0176	–0.0174	0.0002
16	–0.0221	–0.0228	–0.0007	–0.0176	–0.0179	–0.0003
17	–0.0221	–0.0229	–0.0008	–0.0176	–0.0179	–0.0003
18	–0.0221	–0.0228	–0.0007	–0.0176	–0.0179	–0.0003
7	–0.0244	–0.0241	0.0003	–0.0238	–0.0209	0.0029
19	–0.0287	–0.0270	0.0017	–0.0273	–0.0224	0.0049
10	–0.0269	–0.0239	0.0030	–0.0250	–0.0229	0.0021
14	–0.0287	–0.0270	0.0017	–0.0273	–0.0239	0.0034
8	–0.0241	–0.0241	0.0000	–0.0217	–0.0230	–0.0013
13	–0.0272	–0.0270	0.0002	–0.0246	–0.0239	0.0007
Δ*E*_SO_^SIC^(TS–R)			0.0055			0.0131

a PZSIC: columns 2 and 3. LSIC: columns 5 and 6. Columns 4 and 7 give the difference, Δ*U*_*i*_^SIC^(TS–R), for PZSIC and LSIC, respectively. The labels in column 1 are the same as those used in Figure 2. The sum of the differences is shown in the last row. (ii) Same as in (i), but for the spectator orbitals (SOs). The labels in column 1 are the same as those used in Figure S1.

In Table 1, it can be seen that Δ*E*_PO_^SIC^ is dominated by FLO 15, with Δ*U*_15_^SIC^(TS–R) = 18.4 mhartree. FLO 15 evolves from a C–H bond orbital in the reactants to an O–H bond orbital in the products. It has characteristics of both in the transition state and is delocalized over all three atoms (Figure 2b). The value of *U*_*i*_^SIC^ in PZSIC–LSDA is generally negative, as the magnitude of self-Coulomb correction (negative) is nearly always larger than that of the self-exchange-correlation correction (positive). But the magnitude of the self-Coulomb term decreases with increasing delocalization of the orbital. Thus, Δ*U*_*i*_^SIC^ > 0 implies that the transition state orbital is more delocalized than the corresponding orbital in the reactants/products. The large positive value of Δ*U*_15_^SIC^ in Table 1 therefore reflects the change from the relatively compact sp^3^ bonding FLO in the reactants to the strongly delocalized stretched-bond FLO in the transition state.

Δ*U*_*i*_^SIC^ is also positive for the other POs, indicating that they are also more delocalized in the transition state. This can be understood by noting that FLO 2 evolves from a C–H bond orbital in the reactants to an unpaired radical orbital in the products, with some characteristics of both in the transition state. But the relevant C–H bond is elongated in the transition state, making FLO 2 more delocalized and leading to a positive Δ*U*_*i*_^SIC^. Similar considerations hold for FLO 9. For the SOs, most of the values of Δ*U*_*i*_^SIC^ are small in magnitude and are both positive and negative, leading to some cancellation in Δ*E*_SO_^SIC^. The average magnitude of Δ*U*_*i*_^SIC^ from POs and SOs on a per orbital basis for this reaction is 7.3 and 0.64 mhartree, respectively, so that the contribution of an average PO to the barrier correction is an order of magnitude larger than for an average SO. Other reactions where the hypothesis is satisfied have similar features (Table S5).

### 3.3 Example 2: A Reaction Where the Hypothesis Is Not Followed

**Figure 3:**
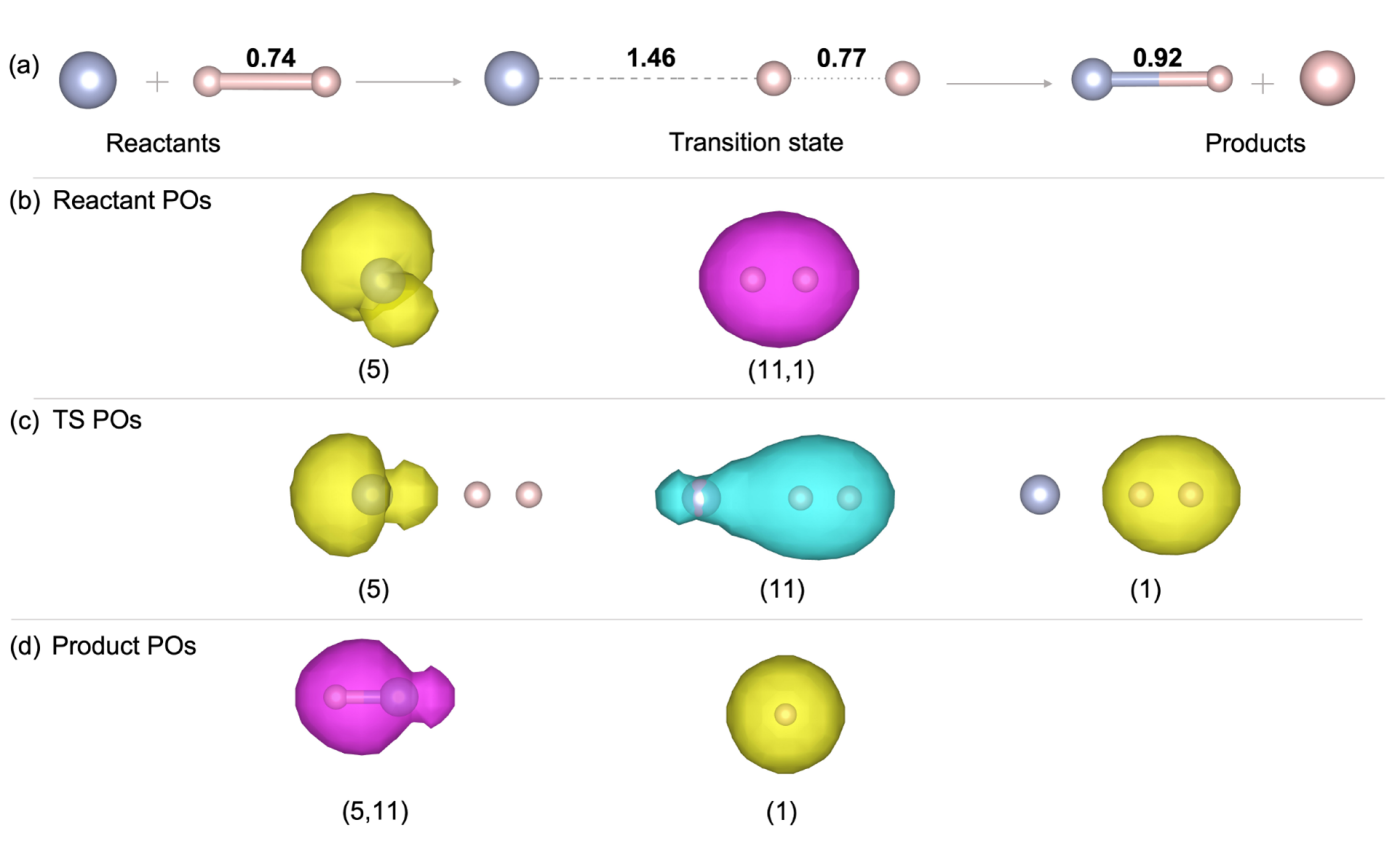
(a) Reaction scheme of T9, F + H_2_ → HF + H. Bond lengths of the active bonds are shown in Å. (b–d) Isosurface plots of PO densities for the reactants (b), the transition state (c), and the products (d). All isosurface values are 0.001/bohr^2^. Color: H (pink), F (blue), unpolarized POs (magenta), up-spin POs (yellow), and down-spin POs (cyan) The PO labels are the same as in Table 2. The orbital indices shown here were automatically generated by the FLOSIC code for the TS and manually mapped to the corresponding orbitals for R and P. A pair of indices, e.g., (11, 1), denotes a pair of spin-up and spin-down orbitals.

In the T9(R) reaction F + H_2_ ←HF + H the H–F bond breaks, allowing the H atoms to form H_2_. The POs are the two FLOs corresponding to the HF bond and the originally unpaired FLO of the H atom. Isosurface plots of the PO densities are given in Figure 3. Note that the unpaired FLO on the F atom must be chosen as the PO in Figure 3b. Plots for the SOs are shown in Figure S2. The PO and SO SIC energies for the products and transition state are given in Table 2 along with their differences.

**Table 2:** (i) SIC Energies *U*_*i*_^SIC^ (hartrees) Computed in Both PZSIC and LSIC for the Participant Orbitals (POs) of the Products (P) and Transition State (TS) for the Reaction T9(R) F + H_2_ → HF + H^a^

(i) PO SIC Energies						
PO	*U*_*i*_^SIC^(TS)	*U*_*i*_^SIC^(P)	Δ*U*_*i*_^SIC^(TS–P)	*U*_*i*_^LSIC^(TS)	*U*_*i*_^LSIC^(P)	Δ*U*_*i*_^LSIC^(TS–P)
5	–0.0334	–0.0267	–0.0067	–0.0299	–0.0246	–0.0053
11	–0.0094	–0.0267	0.0173	–0.0098	–0.0246	0.0148
1	–0.0239	–0.0223	–0.0016	–0.0212	–0.0223	0.0011
Δ*E*_PO_^SIC^(TS–P)			0.009			0.0106

(ii) SO SIC Energies						
SO	*U*_*i*_^SIC^(TS)	*U*_*i*_^SIC^(P)	Δ*U*_*i*_^SIC^ (TS–P)	*U*_*i*_^LSIC^ (TS)	*U*_*i*_^LSIC^ (P)	Δ*U*_*i*_^LSIC^(TS–P)
2	–0.3382	–0.3375	–0.0007	–0.2141	–0.2221	0.0080
7	–0.3382	–0.3375	–0.0007	–0.2355	–0.2221	–0.0134
3	–0.0310	–0.0302	–0.0008	–0.0275	–0.0283	0.0008
4	–0.0310	–0.0303	–0.0007	–0.0283	–0.0283	0.0000
6	–0.0310	–0.0303	–0.0007	–0.0274	–0.0283	0.0009
8	–0.0357	–0.0302	–0.0055	–0.0313	–0.0283	–0.0030
9	–0.0356	–0.0303	–0.0053	–0.0311	–0.0283	–0.0028
10	–0.0354	–0.0303	–0.0051	–0.0309	–0.0283	–0.0026
Δ*E*_SO_^SIC^(TS–P)			–0.0195			–0.0121

a PZSIC: columns 2 and 3. LSIC: columns 5 and 6. Columns 4 and 7 give the difference, Δ*U*_*i*_^SIC^(TS–P), for PZSIC and LSIC, respectively. The labels in column 1 are the same as those used in Figure 3. The sum of the differences is shown in the last row. (ii) Same as in (i), but for the spectator orbitals (SOs). The labels in column 1 are the same as those used in Figure S2.

As in the T4(F) reaction, the largest positive value of Δ*U*_*i*_^SIC^, 0.0173 hartree, is for the stretched bond orbital, in this case FLO 11. However, in this reaction the Δ*U*_*i*_^SIC^ values for the remaining POs are negative, giving a smaller net Δ*E*_PO_^SIC^(TS–P) of 0.009 hartree. The SOs are the F orbitals not involved in the HF bond. These also have negative values of Δ*U*_*i*_^SIC^, indicating that they are more localized in the transition state than in the products. This can be understood by noting that the SOs are transitioning from a polar HF bond where the F atom is partly negatively charged in the products to a neutral F atom in the reactants. The SOs are correspondingly less localized in the products than in the transition state and reactants due to the excess negative charge. The result is Δ*E*_SO_^SIC^(TS–P) = −0.0195 hartree with fractional contributions of −0.86 and 1.86 from the POs and SOs, respectively.

Unlike other cases where positive and negative Δ*U*_*i*_^SIC^ values for the SOs cancel to give a smaller Δ*E*_SO_^SIC^, for this reaction all of the SOs have the same sign, giving a large . For the POs, positive and negative Δ*U*_*i*_^SIC^(TS–P) values cancel to give a smaller net Δ*E*_PO_^SIC^(TS–P). The overall Δ*E*^SIC^ for this reaction is negative so the contribution of SIC to the reaction barrier is negative. We note that for this reaction LSDA underestimates the reference barrier height by 8.58 kcal/mol. Thus, the negative contribution of the SIC part of the energy to the barrier height corrects in the wrong direction. However, the barrier computed from PZSIC for T9 is slightly better than the LSDA barrier, with an improvement of 1.2 kcal/mol (see Table S4). The improvement is due to the inclusion of the changes in the barrier coming from the LSDA part of the PZSIC energy (this contribution is termed “density-driven error”; see below). Because the basis of the stretched-bond hypothesis is that the self-interaction correction to the barrier should be positive, the failure of this reaction to satisfy the hypothesis is expected. Similar observations hold for reactions TN1(F) and T8(R) (Table S6). On the other hand, the main stretched bond orbital PO 11 has a large, positive value of Δ*U*_*i*_^SIC^ of 0.0173 hartree or 10.9 kcal/mol. This is more than three times larger in magnitude than for any other orbital. The average absolute SIC contribution from the POs and SOs on a per orbital basis is 8.53 and 2.4 mhartree, respectively.

### 3.4 Comparison of PZSIC, LSIC, and PZSIC@LSDA

#### 3.4.1 Performance of PZSIC and LSIC Considering Only PO Contributions

As illustrated by the examples above, the SIC contribution to the barriers of the POs is typically positive and increases the barrier heights relative to the LSDA prediction, which nearly always brings the prediction into better agreement with the corresponding reference value. This is in keeping with the premise that self-interaction related to stretched bonds causes the underestimation of barriers in LSDA. But in the last section, we showed that there are cases where the overall barrier contribution from SIC is negative, even when LSDA underestimates the barrier height. In the case of T9(R), using only the positive contribution of the POs to the barrier would improve the barrier height prediction from a significant underestimate in LSDA to a slight overestimate in PZSIC. This prompts the question of whether PZSIC and LSIC would give more accurate reaction barrier energies if only the SIC contribution from the POs were considered in all cases. To address this, we calculated the mean absolute errors (MAE) of PZSIC and LSIC energy barriers with respect to the reference barriers including only the contribution of the POs (Figure S5). We find that both PZSIC and LSIC give smaller MAE when all SIC contributions are included (5.52 and 3.53 kcal/mol, respectively) than when only contributions from the POs are included (7.5 and 4.46 kcal/mol, respectively). The SIC contributions of all orbitals, therefore, appear to be important to the prediction of barrier heights. We note that LSIC has a smaller MAE than PZSIC both when the contribution of all orbitals to the barrier correction are included as well as when only contributions from the POs are included.

#### 3.4.2 Performance of PZSIC and LSIC for Predicting Barriers with Large and Small Self-Interaction Errors

Next, we considered how PZSIC and LSIC perform with respect to reference barrier heights depending on the overall size of the SIC contribution to the barrier. For this, we calculated the mean signed errors (MSE) and mean absolute errors (MAE) in the predicted barrier heights for LSDA, PZSIC, and LSIC relative to reference values for reactions where Δ*E*_Total_^SIC^ > and < 10 kcal/mol. The results for the MSE are shown in Figure 1b. For the reactions with large/small self-interaction corrections, LSDA has MSEs of −20.1 and −11.7 kcal/mol, respectively, and MAEs of 20.13 and 11.91 kcal/mol, respectively. These data indicate that the SIC contributions to the barriers are largest for the reactions where LSDA underestimates the barriers the most. The MSEs for PZSIC are 1.72 and −3.62 kcal/mol for the reactions with large/small corrections, respectively. PZSIC slightly overcorrects the barriers in the first set of reactions on average and undercorrects in the second ones. The MAEs for PZSIC are 4.65 and 6.38 kcal/mol, respectively, for the two sets. For LSIC, the MSEs for the two sets are 4.82 and 0.92 kcal/mol, respectively, and the MAEs are 4.90 and 2.15 kcal/mol, respectively. LSIC clearly outperforms PZSIC for reactions where the corrections are small, whereas the two methods perform about equally well when the corrections are large. The raw data corresponding to the results discussed in this subsection are included in Tables S3 and S4.

#### 3.4.3 PZSIC@LSDA Hypothesis Test

The stretched bond hypothesis is not satisfied in a significant fraction of the reactions we considered. The analysis is based on the SIC energies of FLOs obtained in self-consistent FLOSIC calculations, i.e., using self-interaction-corrected electron densities to determine the FLOs. A natural question is whether the stretched-bond character of the density is reduced in the self-consistent FLOSIC calculation compared to an LSDA calculation and whether that leads, in turn, to POs with less stretched bond character and to smaller contributions from the PO in the FLOSIC calculation. To investigate, we computed SIC corrections to the energy barriers using FLOs that were optimized using the self-consistent LSDA densities. (These FLOs generate total densities equal to the self-consistent LSDA densities.) We refer to these as PZSIC@LSDA calculations. We evaluated the stretched bond hypothesis for PZSIC@LSDA for a subset of 10 HT and 8 NHT reactions, corresponding to a total of 33 distinct reaction barriers. The reactions studied are identified in Table S10. We find that the hypothesis is followed for 70% (23 of 33) of these barriers. This is comparable to the 65% of barriers that satisfy the hypothesis in PZSIC. One interesting difference between the PZSIC and PZSIC@LSDA calculations is that there are no barriers with very small or negative Δ*E*_Total_^SIC^ using the PZSIC@LSDA method. The PZSIC@LSDA results for individual reactions that follow and fail the hypothesis are shown in Tables S10 and S11, respectively.

The converse of a PZSIC@LSDA calculation is a LSDA@PZSIC calculation, i.e., a calculation evaluating the LSDA functional using the self-consistent density of the PZSIC calculation. This is similar to density-corrected DFT (DC–DFT) calculations^35−37^ used to explore density-driven errors in DFT. For the LSDA functional, a density-driven error is defined as Δ*E*_d_ = *E*_LSDA_[*n*_exact_] – *E*_LSDA_[*n*_LSDA_], where *n*_exact_ is the exact electron density of a many-electron system and *n*_LSDA_ is the self-consistent LSDA density of the same system. Stated differently, this is the error LSDA makes by being evaluated on an approximate density, instead of the exact density. Because *n*_exact_ is not normally known, the density from a self-consistent Hartree–Fock calculation, *n*_HF_, is often used as a proxy.^35^*n*_HF_ avoids the delocalization present in some DFT calculations due to electron self-interaction. Mishra et al.^19^ explored density-driven error in PZSIC–LSDA calculations of the BH76 reaction barriers by using the nominally self-interaction-free self-consistent PZSIC density *n*_PZSIC_ instead of *n*_HF_ as the proxy for *n*_exact_ and evaluated the LSDA@PZSIC energy of the TS, P, and R to determine the corresponding barriers. The MAE for the predicted barrier heights using the LSDA@PZSIC energies was reported to be 11.2 kcal/mol as compared to 15.5 kcal/mol for self-consistent LSDA.^19^ This indicates a density-driven error of 4.3 kcal/mol. The remaining error relative to the reference values, 11.2 kcal/mol, is functional-driven. For LSDA, the functional-driven error in the BH76 barrier heights is much larger than the density-driven error.

#### 3.4.4 Why LSIC Predicts Reaction Barriers More Accurately than PZSIC

For the overall set of 66 barriers studied here, LSIC predictions have a MAE of 3.6 kcal/mol when compared to reference values. For PZSIC and LSDA, the MAE are 5.5 and 16.0 kcal/mol, respectively. Both LSIC and PZSIC give much better barrier heights than LSDA, but LSIC is clearly better than PZSIC. This can be understood by considering the performance of the two methods for reaction energies, *E*(P) – *E*(R). The reaction energy can be shown to equal the difference between the forward and reverse reaction barriers. Thus, errors in barrier heights are not independent of reaction energy errors. For the barriers with large SIC corrections (Δ*E*_Total_^SIC^ > 10 kcal/mol), both LSIC and PZSIC have MAE values for the corresponding reaction energies (6.0 and 9.9 kcal/mol, respectively) that are comparable to the LSDA value (8.4 kcal/mol). The MAE in LSIC and PZSIC for the barrier heights for these reactions are also comparable (4.9 and 4.6 kcal/mol, respectively). For the barriers with small SIC corrections (Δ*E*_Total_^SIC^ < 10 kcal/mol), the LSIC MAE for reaction energies (2.3 kcal/mol) is much smaller than for LSDA (6.8 kcal/mol) or PZSIC (5.9 kcal/mol). The corresponding MAE for barrier heights is also much smaller for LSIC (2.2 kcal/mol) than for PZSIC (6.4 kcal/mol). For all reaction barriers studied here, the LSIC and PZSIC MAE for reaction energies are 3.9 and 7.6 kcal/mol, respectively. We note that LSIC is known to improve the description of molecular atomization energies compared to PZSIC.^13^ The reaction energy of a given reaction is equal to the difference in atomization energies of the R and P and LSIC is therefore expected to give more accurate values than PZSIC.

#### 3.4.5 Orbital-by-Orbital Analysis of Outlier Reactions

Mishra et al.^19^ mention two reactions for which the barrier height predictions of PZSIC and LSIC are especially poor. It is of interest to revisit these in the context of the orbital-by-orbital analysis in an attempt to gain further insight into the performance of the SIC methods for these reactions.

T19 is an intramolecular *cis–trans* isomerization reaction of C_5_H_8_ where the positions of two C=C double bonds are shifted along the C chain. The POs in the transition state include orbitals corresponding to all of the C–C bonds. The reference reaction barrier for T19 is 39.7 kcal/mol. LSDA (24.9 kcal/mol) underestimates the barrier by nearly 15 kcal/mol, while both PZSIC (61 kcal/mol) and LSIC (63.7 kcal/mol) overestimate the barrier by more than 20 kcal/mol. The contribution of the DFA part of the PZSIC and LSIC total energy to the barrier height error is −5.2 kcal/mol.^19^ For this reaction the contribution of the POs to the barrier height is nearly identical in PZSIC and LSIC, 0.049 hartree or 30.7 kcal/mol, while the contribution of the SOs is much smaller in both methods, −4.6 kcal/mol for PZSIC and 1.3 kcal/mol for LSIC. The overestimation of the barrier in PZSIC and LSIC thus appears to derive from a too-large upward shift in the PO energies in the transition state. Table S12 shows *U*_*i*_^SIC^ values for reaction T19. Isosurface plots of PO densities are presented in Figure S6.

In the TN1 reaction (H + N_2_O → OH + N_2_), the forward barrier has a reference value of 17.7 kcal/mol. PZSIC (3.5 kcal/mol) underestimates this significantly, while LSIC (18.1 kcal/mol) agrees well. Conversely, the reference value for the reverse barrier is 82.6 kcal/mol and the PZSIC value (84.8 kcal/mol) is close, while LSIC (104.3 kcal/mol) strongly overestimates. The reaction energies for PZSIC and LSIC are similar, −81.3 and −86.2 kcal/mol, respectively, compared to the reference value of −64.9 kcal/mol. So both PZSIC and LSIC predict the energy of the P state to be too deep relative to that of the R state, by roughly similar amounts, 16.4 and 21.3 kcal/mol. But the impact of the reaction energy error is very different in the two cases. In PZSIC, the reaction energy error appears almost entirely in the forward barrier that is too small, implying that the energy of R is too high relative to TS, while the P energy is positioned correctly. In LSIC, the reaction energy error appears in the reverse barrier, making P too deep relative to the TS, while the R energy is positioned properly. In terms of the SIC contributions to the barriers, Δ*E*_Total_^SIC^ for LSIC is larger than for PZSIC for both the forward and reverse barriers by 16.9 and 23.7 kcal/mol, respectively. In both cases, *E*_SO_^SIC^ accounts for most of the difference, 18.4/16.8 kcal/mol for the forward/reverse barriers. This moves the TS up relative to the R and P in LSIC compared to PZSIC.

#### 3.4.6 Why LSIC Follows the Stretched Bond Hypothesis for Fewer Reactions

In Table 1, it can be seen that for the T4(F) reaction, Δ*U*^SIC^ for the most stretched PO (FLO 15) is reduced from 0.018 to 0.012 hartree in going from PZSIC to LSIC. For the remaining PO’s, Δ*U*^SIC^ becomes more positive. The net effect is very little change in Δ*E*_PO_^SIC^. For the SO’s, the shift in Δ*U*^SIC^ from PZSIC to LSIC is mostly positive, making Δ*E*_SO_^SIC^ more positive by 0.0076 hartree. As a result, the fractional contribution of the PO to the total SIC correction is reduced from 0.80 in PZSIC to 0.63 in LSIC, so that the stretched-bond hypothesis is no longer satisfied for T4(F) in LSIC. The PZSIC to LSIC shifts averaged over all the reactions are similar to these. The averaged signed shifts in Δ*E*_PO_^SIC^ and Δ*E*_SO_^SIC^ are −0.001 and 0.007 hartree, respectively. The average absolute shifts are 0.007 and 0.008 hartree. The net effect of the shifts is to make the Δ*E*_PO_^SIC^ contribution to the barrier correction a relatively smaller fraction of the total, resulting in fewer reactions satisfying the stretched bond hypothesis.

## 4 Conclusions

In this work, we explored the origin of one-electron self-interaction error (SIE) in density functional theory predictions of chemical reaction barrier heights. We did this by analyzing data from the Fermi–Löwdin orbital self-interaction correction (FLOSIC) calculations of Mishra et al.^19^ for the reactions of the BH76 benchmark set.^24^ The PZSIC–LSDA and LSIC–LSDA functionals used in those calculations correct for self-interaction on an orbital-by-orbital basis by adding SIC energies for each orbital to the LSDA total energy. Both PZSIC–LSDA and LSIC–LSDA are exact for any one-electron density and can thus be said to be one-electron self-interaction-free. Mishra et al.^19^ found that the mean absolute error in the barrier prediction for the BH76 reactions dropped from 15.5 kcal/mol in LSDA to 5.4 and 3.7 kcal/mol in PZSIC–LSDA and LSIC–LSDA, respectively, by removing SIE. By comparing the SIC energy for an orbital in the reactants/products (R/P) and for the corresponding orbital in the transition state (TS) of a given reaction, we obtain the SIC contribution to the forward/reverse reaction barrier coming from that orbital. We hypothesized that most of the correction for a given barrier would come from the orbitals involved in bond-breaking and bond-making in the TS. We refer to these as participant orbitals (POs). The remaining spectator orbitals (SOs) are not involved in bond rearrangements. We find that the largest source of one-electron SIE is due to the most delocalized PO in the TS. The SIC energy for this stretched bond orbital is considerably more positive than for the corresponding orbital in the R or P. The correction to a barrier from this orbital is therefore positive, i.e., increasing the LSDA prediction for the barrier, which is nearly always too small.

While the average barrier correction associated with a PO is generally much larger than for a SO, the combined contribution of all the SOs can still be significant. For this reason, the stretched-bond hypothesis is not generally valid for either PZSIC or LSIC for the BH76 reactions. For reactions where the total SIC contribution to the barrier is larger than 10 kcal/mol, the hypothesis is valid in 79% of reactions in PZSIC but in only 45% of reactions in LSIC. For the entire set of reactions, the hypothesis holds for 65% of reactions in PZSIC calculations and 52% in LSIC. We find that the overall agreement of the PZSIC and LSIC reaction barriers with reference values is better when contributions from both the POs and SOs are included.

The size of the overall SIC contribution to the barrier is a key indicator in other ways, as well. We found that larger SIC-related barrier corrections in PZSIC are correlated to larger errors in the LSDA barrier predictions and smaller LSDA errors are found when SIC corrections are small. In other words, the size of the SIC contribution to the barrier is properly matched to the size of the LSDA errors. With regard to performance, the MSE and MAE for PZSIC barrier predictions are smaller when the correction due to SIC is large, and vice versa. For LSIC, the opposite is true. LSIC gives a smaller MAE (3.6 kcal/mol) for the barriers overall than PZSIC (5.5 kcal/mol), but especially for the barriers with small SIC corrections. For these, LSIC and PZSIC MAEs are 2.2 and 6.4 kcal/mol, respectively. The better performance of LSIC is shown to be related to more accurate predictions of reaction energies in LSIC than in PZSIC.

Self-interaction corrections can be viewed as addressing either functional-driven or density-driven errors in a density functional approximation (DFA).^35^ A functional-driven error is the error a DFA would make if applied to the exact electron density, whereas density-driven errors arise due to shortcomings in the self-consistent DFA density. The stretched-bond hypothesis investigated in this work relates to functional-driven errors in the LSDA functional. The SIC part of the total energy corrects the underlying DFA, in this case LSDA. As noted above, the largest SIC contribution to barrier height corrections stems from the delocalized stretched bond orbitals. Put simply, there is an energy penalty for the stretched bond POs in the transition state in PZSIC–LSDA calculations.

Very recently, Kaplan et al.^38^ compared density-driven and functional-driven errors for the BH76 set of reaction barrier heights for a variety of DFA’s and using different choices of the proxy for *n*_exact_, the exact density. For LSDA, the Perdew, Burke, and Ernzerhof generalized gradient approximation (PBE)^39^ and the strongly constrained and appropriately normed SCAN meta-GGA,^40^ they also obtained density-driven errors using self-consistent densities from the corresponding PZSIC–DFA calculations as the proxy. From LSDA@PZSIC calculations they find that errors in barrier height predictions are mostly functional-driven, in close agreement with the results of Mishra et al.^19^ On the other hand, from PBE@PZSIC and SCAN@PZSIC calculations they find that the density-driven errors make up a larger fraction of the overall error and the functional-driven error is smaller. This may mean that the SIC contributions to the barriers are smaller in PZSIC–PBE and PZSIC-SCAN, i.e., that the penalty for delocalized orbitals is less pronounced for the more sophisticated functionals. However, Shahi et al.^8^ recently showed that PBE and SCAN make larger errors for the stretched H_2_^+^ bond orbital than LSDA, suggesting that the SIC penalty for delocalized stretched bonds should be larger, rather than smaller, for these functionals. Understanding the SIC barrier corrections for these functionals in more detail using the orbital-by-orbital analysis introduced here would settle this question.

An original motivation for the stretched-bond hypothesis explored in this work was to improve the efficiency of FLOSIC calculations for reaction barrier heights.^21^ If the hypothesis were true, improved barrier heights could be obtained by computing SIC energies only for the small number of POs in a reaction, rather than for all of the orbitals, possibly decreasing the computational cost of the corrections significantly.^21^ While the hypothesis is not strictly satisfied for all BH76 reactions, the results presented here do show that SOs that are relatively unchanged during a reaction do not contribute to the net barrier correction. The BH76 reactions involve very small molecules. In reactions involving larger molecules, more of the orbitals could be expected to behave as true spectators. Thus, approaches that apply SIC only to active regions of a molecule could deliver the relevant corrections much more efficiently.

## Supporting Information Available

Tables for the list of NHT and HT reactions with active bonds in forward and reverse barriers, list of reactions with large and small SIC contributions to the barriers, list of reactions that follow and fail the stretched-bond hypothesis for PZSIC energy, isosurface plots of SO densities for T4(F) and T9(R) reaction, stretched-bond hypothesis analysis for TN19 reaction, list of reactions that follow and fail the hypothesis for LSIC energy, list of reactions that follow and fail the hypothesis for PZSIC@LSDA energy, plot for MAE with respect to the reference value for LSDA, PZSIC, LSIC, PZSIC(PO), and LSIC(PO), isosurface plots of PO densities for T19 reaction, tables with difference in SIC energies of POs and SOs for PZSIC and LSIC energies (PDF)
