# Supplementary material for: How Do Self-Interaction Errors Associated with Stretched Bonds Affect Barrier Height Predictions?
Source: J Phys Chem A. 2023 Feb 14;127(7):1750–9. doi: 10.1021/acs.jpca.2c07894 (PMC10032567; doi:10.1021/acs.jpca.2c07894)
Supplement: Supplementary file 1 — jp2c07894_si_001.pdf [file jp2c07894_si_001.pdf]

Supporting Information: How Do Self-Interaction Errors Associated With Stretched Bonds Affect Barrier Height Predictions?

Priyanka B. Shukla,[†] Prakash Mishra,[‡] Tunna Baruah,[¶,‡] Rajendra R. Zope,[¶,‡] Koblar A. Jackson,[*,§] and J. Karl Johnson[*,†]

†*Department of Chemical & Petroleum Engineering, University of Pittsburgh, Pittsburgh, Pennsylvania 15261, United States*

‡*Computational Science Program, University of Texas at El Paso, El Paso, Texas 79968, United States*

¶*Department of Physics, University of Texas at El Paso, El Paso, Texas 79968, United States*

§*Physics Department and Science of Advanced Materials Program, Central Michigan University, Mount Pleasant, Michigan 48859, United States*

E-mail: jacks1ka@cmich.edu; karlj@pitt.edu

Table S1: Non-hydrogen Transfer (NHT) reactions in the BH76 dataset. Column 3: the first row indicates the reference forward (F) reaction barrier ($\Delta E_{\mathrm{F/R}} = E(\mathrm{TS}) - E(\mathrm{reactants})$) and the second row indicates the reference reverse (R) reaction barrier ($\Delta E_{\mathrm{R}} = E(\mathrm{TS}) - E(\mathrm{products})$). Column 4: reaction energies $\Delta E_{\mathrm{rxn}} = \mathrm{R} - \mathrm{F}$. Columns 5 and 6: active bonds in the forward (F) and reverse (R) reaction barriers. The energies are reported in (kcal/mol).

**Label**	**Reaction**	**Ref** $\Delta E_{\mathrm{F/R}}$	ΔE_{rxn}	**active bonds (F)**	**active bonds (R)**
TN1 (F)	$H + N_2O \rightarrow OH + N_2$	17.7	-64.9	N-O	
(R)		82.6			O-H
TN2 (F)	$H + FH \rightarrow HF + H$	42.1	0	H-F	
(R)		42.1			
TN3 (F)	$H + ClH \rightarrow HCl + H$	17.8	0	H-Cl	
(R)		17.8			
TN4 (F)	$H + FCH_3 \rightarrow HF + CH_3$	30.5	-26.4	C-F	
(R)		56.9			H-F
TN5 (F)	$H + F_2 \rightarrow HF + F$	1.5	-103.3	F-F	
(R)		104.8			H-F
TN6 (F)	$CH_3 + FCl \rightarrow CH_3F + Cl$	7.1	-52.7	F-Cl	
(R)		59.8			C-F
TN7 (F)	$F^- + CH_3F \rightarrow FCH_3 + F^-$	-0.6	0	C-F	
(R)		-0.6			
TN8 (F)	$F^- \ldots CH_3F \rightarrow FCH_3 \ldots F^-$	13.4	0	C-F	
TN9 (F)	$Cl^- + CH_3Cl \rightarrow ClCH_3 + Cl^-$	2.5	0	C-Cl	
(R)		2.5			
TN10 (F)	$Cl^- \ldots CH_3Cl \rightarrow ClCH_3 \ldots Cl^-$	13.5	0	C-Cl	
(R)		13.5		C-Cl	
TN11 (R)	$F^- + CH_3Cl \rightarrow FCH_3 + Cl^-$	19.8			C-F
TN12 (F)	$F^- \ldots CH_3Cl \rightarrow FCH_3 \ldots Cl^-$	3.5	-26.1	C-Cl	
(R)		29.6			C-F
TN13 (R)	$OH^- + CH_3F \rightarrow HOCH_3 + F^-$	17.6			C-O
TN14 (F)	$OH- \ldots CH_3F \rightarrow HOCH_3 \ldots F^-$	11	-36.7	C-F	
(R)		47.7			C-O
TN15 (F)	$H + N_2 \rightarrow HN_2$	14.6	3.7	N-N	
(R)		10.9			N-H
TN16 (F)	$H + CO \rightarrow HCO$	3.2	-19.6	C-O	
(R)		22.8			H-C
TN17 (F)	$H + C_2H_4 \rightarrow CH_3CH_2$	2	-40	C-C	
(R)		42			C-H
TN18 (F)	$CH_3 + C_2H_4 \rightarrow CH_3CH_2CH_2$	6.4	-26.6	C-C	
(R)		33			C-C
TN19(F)	$HCN \rightarrow HNC$	48.1	15.1	H-C	
(R)		33			N-H

Table S2: Hydrogen Transfer (HT) reactions in the BH76 dataset. Column 3: the first row indicates the reference forward (F) reaction barrier ($\Delta E_{\mathrm{F/R}} = E(\mathrm{TS}) - E(\mathrm{reactants})$) and the second row indicates the reference reverse (R) reaction barrier ($\Delta E_{\mathrm{R}} = E(\mathrm{TS}) - E(\mathrm{products})$). Column 4: reaction energies $\Delta E_{\mathrm{rxn}} = \mathrm{R} - \mathrm{F}$. Columns 5 and 6: active bonds in the forward (F) and reverse (R) reaction barriers. The energies are reported in (kcal/mol).

Label	Reaction	Ref$\Delta E_{\mathrm{F/R}}$	ΔE_{rxn}	active bonds (F)	active bonds (R)
T1(F)	$H + HCl \to H_2 + Cl$	6.1	-1.9	H-Cl	
(R)		8			H-H
T2(F)	$OH + H_2 \to H_2O + H$	5.2	-16.4	H-H	
(R)		21.6			O-H
T3(F)	$CH_3 + H_2 \to CH_4 + H$	11.9	-3.1	H-H	
(R)		15			C-H
T4(F)	$OH + CH_4 \to H_2O + CH_3$	6.3	-13.2	C-H	
(R)		19.5			O-H
T5(F)	$H + H_2 \to H_2 + H$	9.7	0	H-H	
(R)		9.7			
T6(F)	$OH + NH_3 \to H_2O + NH_2$	3.4	-10.3	N-H	
(R)		13.7			O-H
T7(F)	$HCl + CH_3 \to CH_4 + Cl$	1.8	-5	H-Cl	
(R)		6.8			C-H
T8(F)	$OH + C_2H_6 \to H_2O + C_2H_5$	3.5	-16.9	C-H	
(R)		20.4			O-H
T9(F)	$F + H_2 \to HF + H$	1.6	-32.2	H-H	
(R)		33.8			H-F
T10(F)	$O + CH_4 \to OH + CH_3$	14.4	5.5	C-H	
(R)		8.9			O-H
T11(F)	$H + PH_3 \to H_2 + PH_2$	2.9	-21.8	P-H	
(R)		24.7			H-H
T12(F)	$H + HO \to H_2 + O$	10.9	-2.3	O-H	
(R)		13.2			H-H
T13(F)	$H + H_2S \to H_2 + HS$	3.9	-13.3	H-S	
(R)		17.2			H-H
T14(F)	$O + HCl \to OH + Cl$	10.4	0.5	H-Cl	
(R)		9.9			O-H
T15(F)	$CH_3 + NH_2 \to CH_4 + NH$	8.9	-13.1	N-H	
(R)		22			C-H
T16(F)	$C_2H_5 + NH_2 \to C_2H_6 + NH$	9.8	-9.6	N-H	
(R)		19.4			C-H
T17(F)	$NH_2 + C_2H_6 \to NH_3 + C_2H_5$	11.3	-6.5	C-H	
(R)		17.8			N-H
T18(F)	$NH_2 + CH_4 \to NH_3 + CH_3$	13.9	-3	C-H	
(R)		16.9			N-H
T19(F)	$s-transcis-C_5H_8 \to s-transcis-C_5H_8$	39.7	0	C-H	
(R)		39.7			

Table S3: Reactions with $\Delta E^{\rm SIC}_{\rm Total} > 10$ kcal/mol as calculated in PZSIC. The table includes the fractional contribution to the reaction barrier correction from the POs for PZSIC and LSIC energies, errors with respect to the reference energy for LDA, PZSIC and LSIC in kcal/mol, average fraction contribution from the POs, and the mean signed error (MSE). The standard deviation is given at the bottom of the table. Two tailed t-test values were calculated by using two sets: PZSIC ($\Delta E^{\rm SIC}_{\rm Total} > 10$ kcal/mol) and ($\Delta E^{\rm SIC}_{\rm Total} < 10$ kcal/mol). The reactions with PZSIC $\Delta E^{\rm SIC}_{\rm Total} < 10$ kcal/mol are shown in Table S4.

Reactions	Frac (PO) (PZSIC)	Frac (PO) (LSIC)	LDA–Ref	PZSIC–Ref	LSIC–Ref
TN1 (R) $H + N_2O \rightarrow OH + N_2$	0.91	0.62	-48.89	2.19	21.66
TN2 (F) $H + FH \rightarrow HF + H$	1.02	0.69	-23.66	-4.36	-1.27
TN3 (F) $H + ClH \rightarrow HCl + H$	0.90	0.76	-14.80	0.71	2.73
TN4 (F) $H + FCH_3 \rightarrow HF + CH_3$	1.12	0.77	-17.71	13.09	3.66
TN4 (R) $H + FCH_3 \rightarrow HF + CH_3$	1.41	0.56	-25.00	-0.28	11.10
TN5 (R) $H + F_2 \rightarrow HF + F$	1.88	0.85	-35.63	-0.27	12.80
TN6 (F) $CH_3 + FCl \rightarrow CH_3F + Cl$	1.01	0.19	-19.47	1.63	3.75
TN6 (R) $CH_3 + FCl \rightarrow CH_3F + Cl$	1.34	0.83	-19.66	9.37	4.52
TN7 (F) $F^- + CH_3F \rightarrow FCH_3 + F^-$	1.08	0.65	-11.68	2.66	6.63
TN9 (F) $Cl^- + CH_3Cl \rightarrow ClCH_3 + Cl^-$	1.06	0.70	-10.65	3.27	3.87
TN11 (R) $F^- + CH_3Cl \rightarrow FCH_3 + Cl^-$	0.32	0.20	-10.81	6.16	4.67
TN12 (R) $F^- ... CH_3Cl \rightarrow FCH_3 ... Cl^-$	0.33	0.00	-8.80	6.45	0.42
TN13 (R) $OH^- + CH_3F \rightarrow HOCH_3 + F^-$	1.33	0.82	-11.19	5.18	8.06
TN14 (F) $OH^- ... CH_3F \rightarrow HOCH_3 ... F^-$	0.99	1.18	-9.19	3.67	0.34
TN14 (R) $OH^- ... CH_3F \rightarrow HOCH_3 ... F^-$	1.68	7.62	-0.37	12.49	2.92
TN15 (R) $H + N_2 \rightarrow HN_2$	1.23	1.16	-1.51	16.43	10.80
T1 (R) $H + HCl \rightarrow H_2 + Cl$	0.66	0.69	-17.21	-7.02	1.12
T2 (F) $OH + H_2 \rightarrow H_2O + H$	0.74	0.65	-23.91	-4.96	2.80
T4 (F) $OH + CH_4 \rightarrow H_2O + CH_3$	0.80	0.63	-23.71	-1.89	1.22
T6 (F) $OH + NH_3 \rightarrow H_2O + NH_2$	0.87	0.63	-27.64	0.57	2.50
T6 (R) $OH + NH_3 \rightarrow H_2O + NH_2$	1.11	0.71	-24.41	-3.24	3.48
T7 (R) $HCl + CH_3 \rightarrow CH_4 + Cl$	0.84	0.74	-15.97	-2.23	0.62
T8 (F) $OH + C_2H_6 \rightarrow H_2O + C_2H_5$	0.77	0.60	-24.65	-1.58	2.30
T9 (F) $F + H_2 \rightarrow HF + H$	0.67	0.53	-25.32	-4.52	2.15
T10 (F) $O + CH_4 \rightarrow OH + CH_3$	0.81	0.63	-25.61	-1.37	1.70
T12 (R) $H + HO \rightarrow H_2 + O$	0.73	0.61	-27.28	-4.67	1.60
T14 (F) $O + HCl \rightarrow OH + Cl$	0.84	0.56	-35.53	-1.89	2.31
T14 (R) $O + HCl \rightarrow OH + Cl$	1.01	0.75	-28.37	-2.40	1.51
T15 (R) $CH_3 + NH_2 \rightarrow CH_4 + NH$	0.88	0.67	-19.57	-2.45	2.30
T16 (R) $C_2H_5 + NH_2 \rightarrow C_2H_6 + NH$	0.83	0.63	-20.65	-2.15	3.30
T17 (F) $NH_2 + C_2H_6 \rightarrow NH_3 + C_2H_5$	0.81	0.62	-20.79	-1.93	3.80
T18 (F) $NH_2 + CH_4 \rightarrow NH_3 + CH_3$	0.77	0.64	-19.90	-1.08	3.31
T19 (F) s−trans−cis−$C_5H_8 \rightarrow$ s−trans−cis−C_5H_8	1.18	1.00	-14.76	21.32	26.60
Average fraction	0.97	0.88			
MSE			-20.13	1.72	4.83
MAE			20.13	4.65	4.90
Standard deviation	0.32	1.21	9.60	6.45	5.82
T-Test	0.24	0.92	0.00016	0.00169	0.00145

Table S4: Reactions with $\Delta E^{\text{SIC}}_{\text{Total}} < 10$ kcal/mol as calculated from PZSIC. The table includes fractional contribution to the reaction barrier correction from the POs for PZSIC and LSIC energies, errors with respect to the reference energy for LDA, PZSIC and LSIC in kcal/mol, the average fraction contribution from the POs, and the mean signed error (MSE). The standard deviation is given at the bottom of the table. The reactions with PZSIC $\Delta E^{\text{SIC}}_{\text{Total}} > 10$ kcal/mol are shown in Table S3.

Reactions	Frac (PO) (PZSIC)	Frac (PO) (LSIC)	LDA–Ref	PZSIC–Ref	LSIC–Ref
TN1 (F) $H + N_2O \rightarrow OH + N_2$	-1.02	3.42	-15.35	-14.19	0.35
TN5 (F)$H + F_2 \rightarrow HF + F$	-3.04	0.30	-17.42	0.15	6.00
TN8 (F)$F^- ... CH_3F \rightarrow FCH_3 ... F^-$	1.20	1.08	-7.50	4.24	1.33
TN10 (F) $Cl^- ... CH_3Cl \rightarrow ClCH_3 ... Cl^-$	1.42	1.11	-7.83	2.31	-0.57
TN12 (F) $F^- ... CH_3Cl \rightarrow FCH_3 ... Cl^-$	1.78	1.54	-5.15	2.04	0.35
TN15 (F) $H + N_2 \rightarrow HN_2$	2.86	1.01	-16.76	-5.54	2.10
TN16 (F) $H + CO \rightarrow HCO$	0.78	0.81	-10.77	-4.22	1.58
TN16 (R) $H + CO \rightarrow HCO$	0.83	1.08	3.45	13.86	10.58
TN17 (F) $H + C_2H_4 \rightarrow CH_3CH_2$	1.09	0.90	-7.35	-2.82	4.03
TN17 (R) $H + C_2H_4 \rightarrow CH_3CH_2$	2.12	2.15	-2.51	4.56	0.69
TN18 (F) $CH_3 + C_2H_4 \rightarrow CH_3CH_2CH_2$	0.59	0.52	-12.12	-7.40	7.14
TN18 (R) $CH_3 + C_2H_4 \rightarrow CH_3CH_2CH_2$	2.12	-0.61	0.08	9.94	-4.50
TN19 (F) $HCN \rightarrow HNC$	5.13	3.17	-3.55	2.80	3.11
TN19 (R) $HCN \rightarrow HNC$	2.65	1.86	-2.57	5.64	5.12
T1 (F) $H + HCl \rightarrow H_2 + Cl$	1.30	1.11	-9.49	-2.95	-1.46
T2 (R) $OH + H_2 \rightarrow H_2O + H$	4.49	2.07	-10.45	-3.99	-1.10
T3 (F) $CH_3 + H_2 \rightarrow CH_4 + H$	0.71	0.70	-17.24	-12.17	-0.62
T3 (R) $CH_3 + H_2 \rightarrow CH_4 + H$	1.30	1.37	-10.07	-0.59	-1.40
T4 (R) $OH + CH_4 \rightarrow H_2O + CH_3$	18.75	0.81	-17.32	-12.40	-0.16
T5 (F) $H + H_2 \rightarrow H_2 + H$	1.00	1.00	-12.34	-4.09	-1.00
T7 (F) $HCl + CH_3 \rightarrow CH_4 + Cl$	0.84	0.57	-15.43	-9.77	-1.26
T8 (R) $OH + C_2H_6 \rightarrow H_2O + C_2H_5$	-0.52	0.87	-15.60	-13.69	-0.04
T9 (R) $F + H_2 \rightarrow HF + H$	-0.86	-7.07	-8.58	-7.41	-1.80
T10 (R) $O + CH_4 \rightarrow OH + CH_3$	1.25	0.75	-17.92	-9.43	-0.98
T11 (F) $H + PH_3 \rightarrow H_2 + PH_2$	0.93	0.88	-10.20	-2.43	-1.00
T11 (R) $H + PH_3 \rightarrow H_2 + PH_2$	0.72	0.75	-34.59	-7.09	4.10
T12 (F) $H + HO \rightarrow H_2 + O$	1.42	1.24	-9.29	-1.25	-1.90
T13 (F) $H + H_2S \rightarrow H_2 + HS$	0.98	0.95	-10.64	-2.60	-1.70
T13 (R) $H + H_2S \rightarrow H_2 + HS$	0.63	0.70	-17.05	-7.52	-0.90
T15 (F) $CH_3 + NH_2 \rightarrow CH_4 + NH$	0.78	0.66	-17.31	-7.18	1.25
T16 (F) $C_2H_5 + NH_2 \rightarrow C_2H_6 + NH$	0.67	0.66	-15.54	-8.25	1.46
T17 (R) $NH_2 + C_2H_6 \rightarrow NH_3 + C_2H_5$	1.30	0.71	-14.87	-10.11	0.64
T18 (R) $NH_2 + CH_4 \rightarrow NH_3 + CH_3$	0.90	0.67	-16.83	-7.89	0.80
Average fraction	1.67	0.84			
MSE			-11.70	-3.62	0.92
MAE			11.91	6.38	2.15
Standard deviation	3.33	1.58	6.88	6.59	2.99

Table S5: The set of BH76 reactions that follow the hypothesis for PZSIC calculations. The forward (F) and reverse (R) barrier SIC for the POs and SOs and the total SIC energies are reported in Ha. Frac (PO) is calculated as the ratio of $\Delta E^{\mathrm{SIC}}_{\mathrm{PO}}/\Delta E^{\mathrm{SIC}}_{\mathrm{Total}}$. Similarly, Frac (SO) is calculated as the ratio of $\Delta E^{\mathrm{SIC}}_{\mathrm{SO}}/\Delta E^{\mathrm{SIC}}_{\mathrm{Total}}$.

Label	Reaction	$\Delta E^{\mathrm{SIC}}_{\mathrm{PO}}$	Frac(PO)	$\Delta E^{\mathrm{SIC}}_{\mathrm{SO}}$	Frac(SO)	$\Delta E^{\mathrm{SIC}}_{\mathrm{Total}}$
TN1 (R)	$H + N_2O \rightarrow OH + N_2$	0.0399	0.91	0.0039	0.09	0.0438
TN2 (F)	$H + FH \rightarrow HF + H$	0.0255	1.02	-0.0005	-0.02	0.0250
TN3 (F)	$H + ClH \rightarrow HCl + H$	0.0172	0.90	0.0020	0.10	0.0192
TN4 (F)	$H + FCH_3 \rightarrow HF + CH_3$	0.0399	1.12	-0.0043	-0.12	0.0356
TN6 (F)	$CH_3 + FCl \rightarrow CH_3F + Cl$	0.0176	1.01	-0.0002	-0.01	0.0174
TN7 (F)	$F^- + CH_3F \rightarrow FCH_3 + F^-$	0.0207	1.08	-0.0015	-0.08	0.0192
TN8 (F)	$F^- ... CH_3F \rightarrow FCH_3 ... F^-$	0.0192	1.20	-0.0032	-0.20	0.016
TN9 (F)	$Cl^- + CH_3Cl \rightarrow ClCH_3 + Cl^-$	0.0202	1.06	-0.0011	-0.06	0.0191
TN13 (R)	$OH^- + CH_3F \rightarrow HOCH_3 + F^-$	0.0294	1.33	-0.0073	-0.33	0.0221
TN14 (F)	$OH^- ... CH_3F \rightarrow HOCH_3 ... F^-$	0.0174	0.99	0.0002	0.01	0.0176
TN15 (R)	$H + N_2 \rightarrow HN_2$	0.0301	1.23	-0.0057	-0.23	0.0244
TN16 (F)	$H + CO \rightarrow HCO$	0.0052	0.78	0.0015	0.22	0.0067
TN16 (R)	$H + CO \rightarrow HCO$	0.0124	0.83	0.0025	0.17	0.0149
TN17 (F)	$H + C_2H_4 \rightarrow CH_3CH_2$	0.0058	1.09	-0.0005	-0.09	0.0053
T1 (F)	$H + HCl \rightarrow H_2 + Cl$	0.0107	1.30	-0.0025	-0.30	0.0082
T2 (F)	$OH + H_2 \rightarrow H_2O + H$	0.0162	0.74	0.0058	0.26	0.0220
T3 (F)	$CH_3 + H_2 \rightarrow CH_4 + H$	0.0035	0.71	0.0014	0.29	0.0049
T3 (R)	$CH_3 + H_2 \rightarrow CH_4 + H$	0.0174	1.30	-0.0040	-0.30	0.0134
T4 (F)	$OH + CH_4 \rightarrow H_2O + CH_3$	0.0219	0.80	0.0055	0.20	0.0274
T5 (F)	$H + H_2 \rightarrow H_2 + H$	0.0115	1.00	0.0000	0.00	0.0115
T6 (F)	$OH + NH_3 \rightarrow H_2O + NH_2$	0.0301	0.87	0.0045	0.13	0.0346
T6 (R)	$OH + NH_3 \rightarrow H_2O + NH_2$	0.0264	1.11	-0.0026	-0.11	0.0238
T7 (F)	$HCl + CH_3 \rightarrow CH_4 + Cl$	0.0056	0.84	0.0011	0.16	0.0067
T7 (R)	$HCl + CH_3 \rightarrow CH_4 + Cl$	0.0195	0.84	0.0036	0.16	0.0231
T8 (F)	$OH + C_2H_6 \rightarrow H_2O + C_2H_5$	0.0203	0.77	0.0062	0.23	0.0265
T9 (F)	$F + H_2 \rightarrow HF + H$	0.0132	0.67	0.0064	0.33	0.0196
T10 (F)	$O + CH_4 \rightarrow OH + CH_3$	0.0257	0.81	0.0059	0.19	0.0316
T10 (R)	$O + CH_4 \rightarrow OH + CH_3$	0.0105	1.25	-0.0021	-0.25	0.0084
T11 (F)	$H + PH_3 \rightarrow H_2 + PH_2$	0.0090	0.93	0.0007	0.07	0.0097
T11 (R)	$H + PH_3 \rightarrow H_2 + PH_2$	0.0062	0.72	0.0024	0.28	0.0086
T12 (R)	$H + HO \rightarrow H_2 + O$	0.0210	0.73	0.0076	0.26	0.0286
T13 (F)	$H + H_2S \rightarrow H_2 + HS$	0.0104	0.98	0.0002	0.02	0.0106
T14 (F)	$O + HCl \rightarrow OH + Cl$	0.0268	0.84	0.0052	0.16	0.0320
T14 (R)	$O + HCl \rightarrow OH + Cl$	0.0255	1.01	-0.0003	-0.01	0.0252
T15 (F)	$CH_3 + NH_2 \rightarrow CH_4 + NH$	0.0089	0.78	0.0025	0.22	0.0114
T15 (R)	$CH_3 + NH_2 \rightarrow CH_4 + NH$	0.0205	0.88	0.0029	0.12	0.0234
T16 (F)	$C_2H_5 + NH_2 \rightarrow C_2H_6 + NH$	0.0058	0.67	0.0029	0.33	0.0087
T16 (R)	$C_2H_5 + NH_2 \rightarrow C_2H_6 + NH$	0.0206	0.83	0.0042	0.17	0.0248
T17 (F)	$NH_2 + C_2H_6 \rightarrow NH_3 + C_2H_5$	0.0202	0.81	0.0047	0.18	0.0249
T17 (R)	$NH_2 + C_2H_6 \rightarrow NH_3 + C_2H_5$	0.0060	1.30	-0.0014	-0.30	0.0046
T18 (F)	$NH_2 + CH_4 \rightarrow NH_3 + CH_3$	0.0203	0.77	0.0062	0.23	0.0265
T18 (R)	$NH_2 + CH_4 \rightarrow NH_3 + CH_3$	0.0093	0.90	0.0010	0.10	0.0103
T19 (F)	$s{-}transcis{-}C_5H_8 \rightarrow s{-}transcis{-}C_5H_8$	0.0489	1.18	-0.0074	-0.18	0.0415

Table S6: The set of BH76 reactions that fail the hypothesis for PZSIC calculations. The forward (F) and reverse (R) barrier SIC for the POs and SOs and the total SIC energies are reported in Ha. Frac (PO) is calculated as the ratio of $\Delta E^{\mathrm{SIC}}_{\mathrm{PO}}/\Delta E^{\mathrm{SIC}}_{\mathrm{Total}}$. Similarly, Frac (SO) is calculated as the ratio of $\Delta E^{\mathrm{SIC}}_{\mathrm{SO}}/\Delta E^{\mathrm{SIC}}_{\mathrm{Total}}$.

Label	Reaction	$\Delta E^{\mathrm{SIC}}_{\mathrm{PO}}$	Frac(PO)	$\Delta E^{\mathrm{SIC}}_{\mathrm{SO}}$	Frac(SO)	$\Delta E^{\mathrm{SIC}}_{\mathrm{Total}}$
TN4 (R)	$H + FCH_3 \rightarrow HF + CH_3$	0.0350	1.41	-0.0101	-0.41	0.0249
TN6 (R)	$CH_3 + FCl \rightarrow CH_3F + Cl$	0.0433	1.34	-0.0111	-0.34	0.0322
TN10 (F)	$Cl^- ... CH_3Cl \rightarrow ClCH_3 ... Cl^-$	0.0180	1.42	-0.0053	-0.42	0.0127
TN11 (R)	$F^- + CH_3Cl \rightarrow FCH_3 + Cl^-$	0.0068	0.32	0.0147	0.68	0.0215
TN12 (R)	$F^- ... CH_3Cl \rightarrow FCH_3 ... Cl^-$	0.0058	0.33	0.0118	0.67	0.0176
TN14 (R)	$OH^- ... CH_3F \rightarrow HOCH_3 ... F^-$	0.0280	1.68	-0.0113	-0.68	0.0167
TN18 (F)	$CH_3 + C_2H_4 \rightarrow CH_3CH_2CH_2$	0.0019	0.59	0.0013	0.41	0.0032
T1 (R)	$H + HCl \rightarrow H_2 + Cl$	0.0107	0.66	0.0054	0.33	0.0161
T12 (F)	$H + HO \rightarrow H_2 + O$	0.0197	1.42	-0.0058	-0.42	0.0139
T13 (R)	$H + H_2S \rightarrow H_2 + HS$	0.0081	0.63	0.0049	0.37	0.0130
TN1 (F)	$H + N_2O \rightarrow OH + N_2$	0.0218	-1.02	-0.0431	2.02	-0.0213
TN5 (F)	$H + F_2 \rightarrow HF + F$	-0.0079	-3.04	0.0105	4.04	0.0026
TN5 (R)	$H + F_2 \rightarrow HF + F$	0.0563	1.88	-0.0264	-0.88	0.0299
TN12 (F)	$F^- ... CH_3Cl \rightarrow FCH_3 ... Cl^-$	0.0174	1.78	-0.0076	-0.78	0.0098
TN15 (F)	$H + N_2 \rightarrow HN_2$	0.0212	2.86	-0.0138	-1.86	0.0074
TN17 (R)	$H + C_2H_4 \rightarrow CH_3CH_2$	0.0218	2.12	-0.0115	-1.12	0.0103
TN18 (R)	$CH_3 + C_2H_4 \rightarrow CH_3CH_2CH_2$	0.0286	2.12	-0.0151	-1.12	0.0135
TN19 (F)	$HCN \rightarrow HNC$	0.0246	5.13	-0.0198	-4.13	0.0048
TN19 (R)	$HCN \rightarrow HNC$	0.0212	2.65	-0.0132	-1.65	0.008
T2 (R)	$OH + H_2 \rightarrow H_2O + H$	0.0157	4.49	-0.0122	-3.49	0.0035
T4 (R)	$OH + CH_4 \rightarrow H_2O + CH_3$	0.0075	18.75	-0.0071	-17.75	0.0004
T8 (R)	$OH + C_2H_6 \rightarrow H_2O + C_2H_5$	0.0024	-0.52	-0.007	1.52	-0.0046
T9 (R)	$F + H_2 \rightarrow HF + H$	0.0090	-0.86	-0.0195	1.86	-0.0105

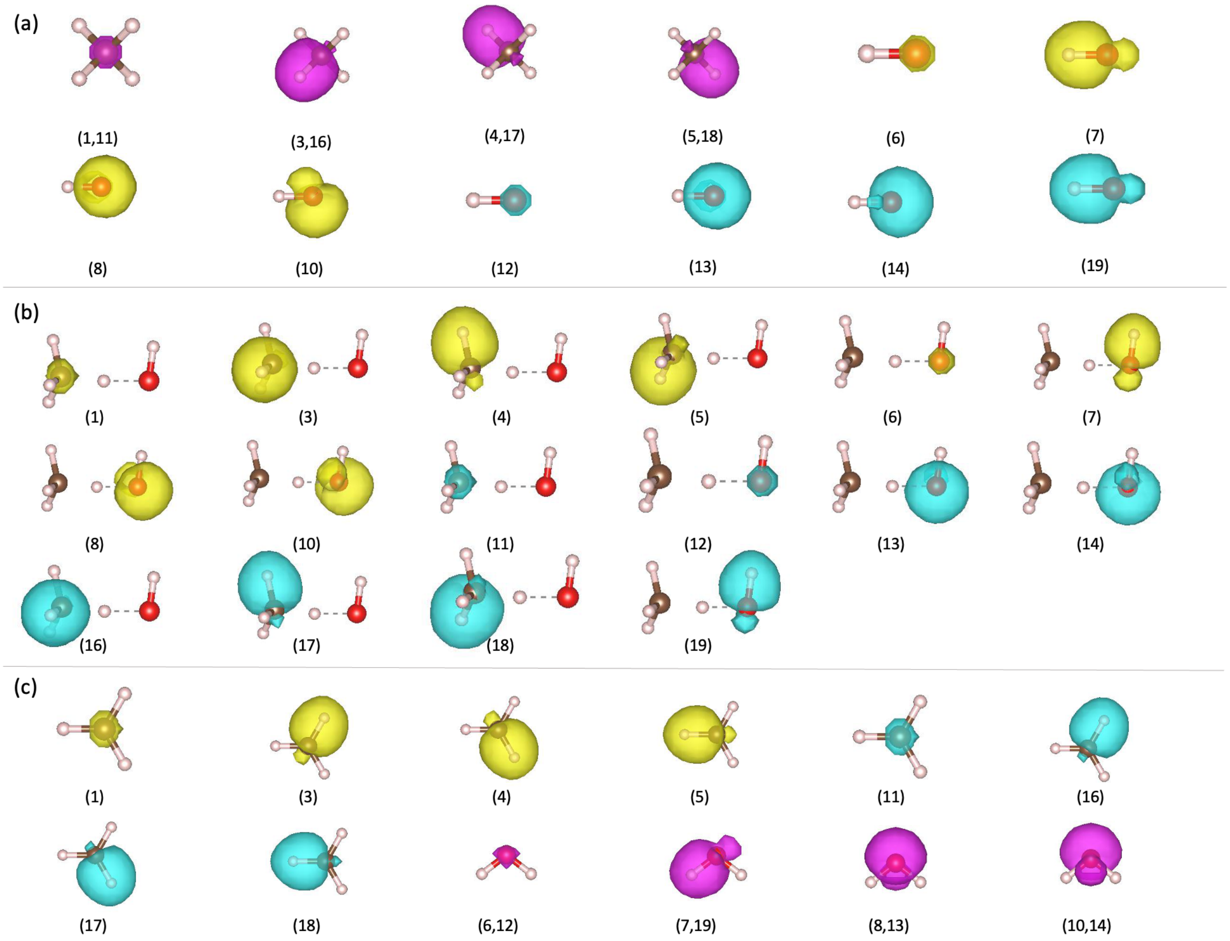

Figure S1: (a-c) Isosurface plots for SO densities of the reactants (a), transition state (b), and products (c) for the reaction T4 OH + $CH_4 \rightarrow H_2O$ + CH_3. All isosurface values are 0.001/$Bohr^2$. Color: C (brown), H (pink), O (red), unpolarized SO (purple), up-spin SO (yellow) and down-spin SO (cyan). The SO labels are the same as in Table 1 of the main text. The orbital indices shown here were automatically generated by the FLOSIC code for the TS and manually mapped to the corresponding orbitals for R and P. A pair of indices, e.g., (1,11), denote a pair spin-up and spin-down orbitals.

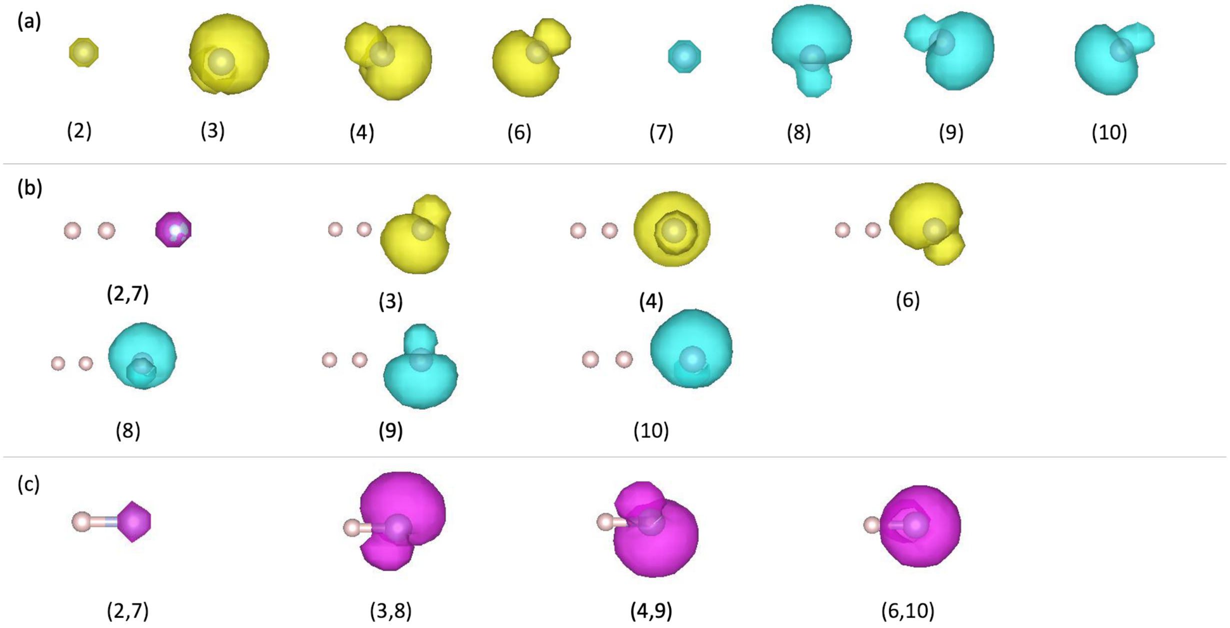

Figure S2: (a-c) Isosurface plots for SO densities of the reactants (a), transition state (b), and products (c) for the reaction T9 $F + H_2 \rightarrow HF + H$. All isosurface values are $0.001/\text{Bohr}^2$. Color: H (pink), F (blue), unpolarized SO (purple), up-spin SO (yellow) and down-spin SO (cyan). The SO labels are the same as in Table 2 of the main text. The orbital indices shown here were automatically generated by the FLOSIC code for the TS and manually mapped to the corresponding orbitals for R and P. A pair of indices, e.g., (2,7), denote a pair spin-up and spin-down orbitals.

TN19 reaction

TN19 (HCN → HNC) is a unimolecular proton transfer reaction. The reaction mechanism is shown in Figure S3. The proton transfer takes place as the C−H bond of HCN breaks leaving the two FLOs of the C−H bond on the C atom as the lone pair FLOs. The H atom is shared with C and N giving a triangular planar structure. The H atom then forms an H−N bond with the lone pair of N to form linear a HNC molecule in the product state.

Each of the three triple bonds (6 FLOs) of the CN bond in HCN are *potential* POs, but the minimal set of participants orbitals (PO) includes only one of the triple bonds (2 FLOs), along with the N lone pair FLOs and the C−H bond pair FLOs. The R, TS, and P POs and SOs are shown in Figures S3 and S4, respectively. In the TS, the two FLOs of one of the triple bonds are delocalized over the H atom. The proton transfer mechanism changes the SIC for all the other SOs going from R to TS to P, leading to a failure of the hypothesis in both directions. The mapping of the R and P POs and SOs with the TS POs and SOs are shown in Table S7. The average absolute SIC contribution on a per-orbital basis in the forward direction is 4.2 mHa and 2.4 mHa, for POs and SOs, respectively. Similarly, the average absolute SIC contribution on a per-orbital basis in the reverse direction is 3.5 mHa and 1.9 mHa for POs and SOs, respectively.

The POs and SOs contribute similar orders of magnitude of SIC energies. Almost equal and opposite $\Delta E^{\mathrm{SIC}}_{\mathrm{PO}}(\mathrm{TS}-\mathrm{R/P})$ and $\Delta E^{\mathrm{SIC}}_{\mathrm{SO}}(\mathrm{TS}-\mathrm{R/P})$, cancel each other to give a small $\Delta E^{\mathrm{SIC}}_{\mathrm{Total}}(\mathrm{TS}-\mathrm{R/P})$ of 0.0048 Ha and 0.008 Ha in the forward and reverse directions, respectively. The small $\Delta E^{\mathrm{SIC}}_{\mathrm{Total}}(\mathrm{TS}-\mathrm{R/P})$ contributes to the failure of the stretched bond hypothesis, giving fractional contributions of 5.13 and -4.13 from the POs and SOs, respectively, in the forward direction, and of 2.65 and -1.65, respectively, in the reverse direction (see Table S6). Many of the other reactions that fail the hypothesis have small $\Delta E^{\mathrm{SIC}}_{\mathrm{Total}}(\mathrm{TS}-\mathrm{R/P})$.

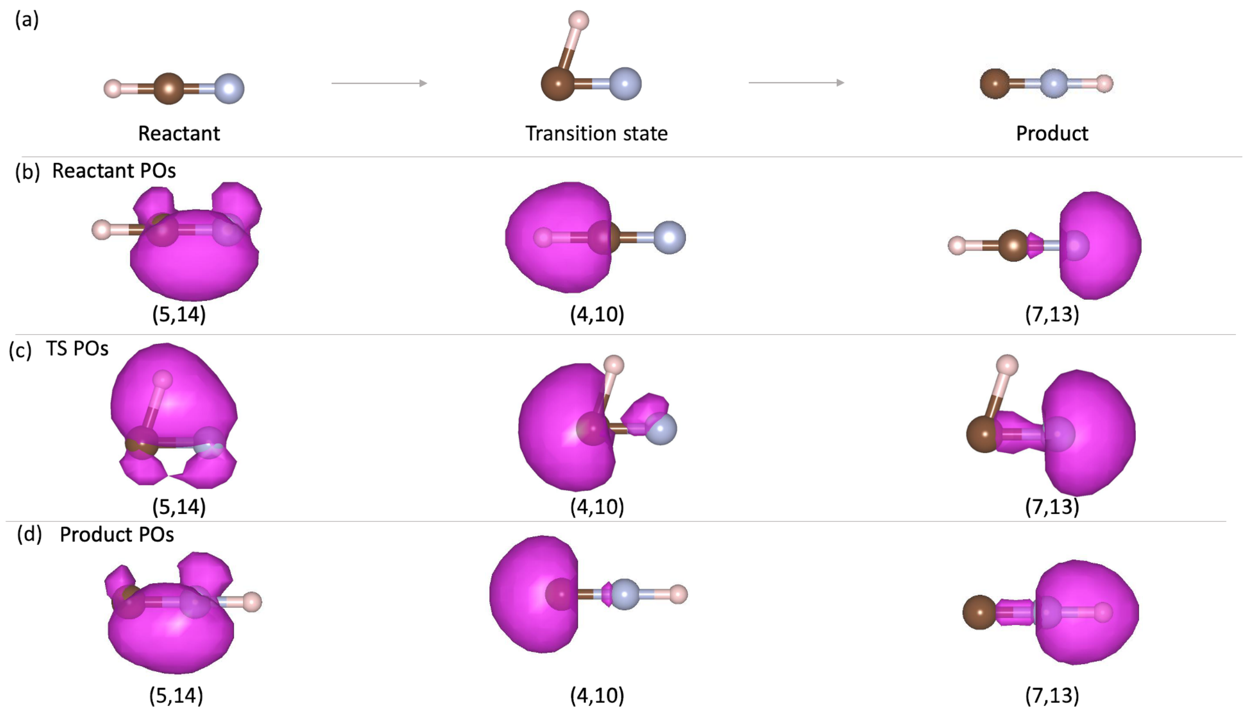

Figure S3: (a) The reaction scheme of TN19 HCN → HNC. (b-d) Isosurface plots for PO densities in the reactant (b), transition state (c), and product (d). All isosurface values are 0.001/Bohr^2. Color: C (brown), H (pink), N (blue), unpolarized PO (purple). The PO labels are the same as in Table S7. The orbital indices shown here were automatically generated by the FLOSIC code for the TS and manually mapped to the corresponding orbitals for R and P.

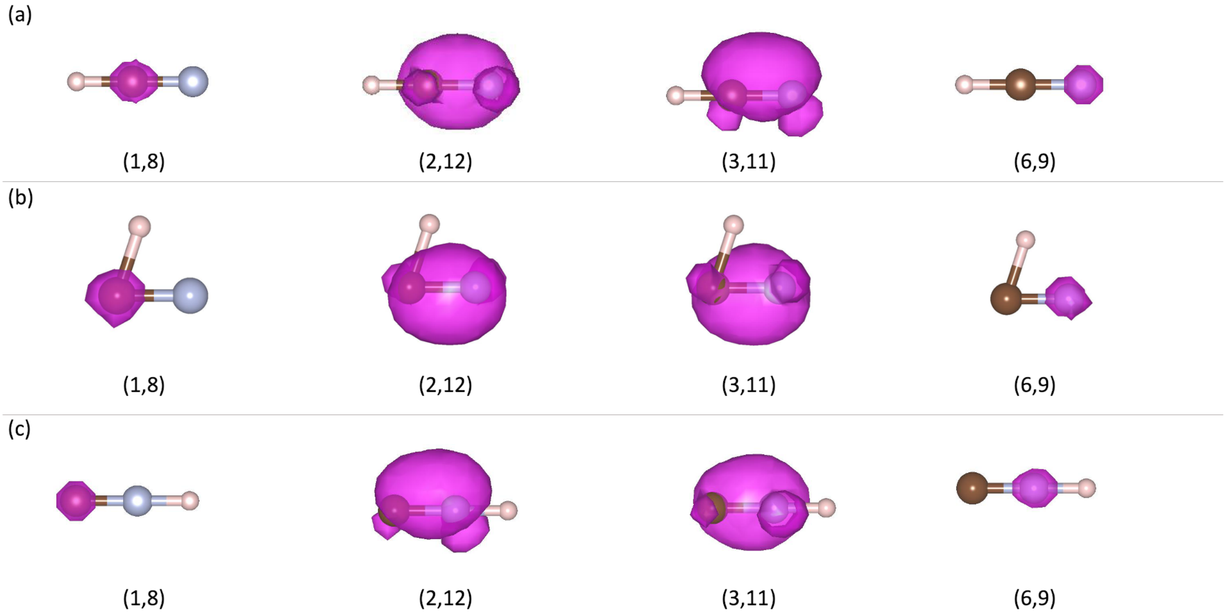

Figure S4: (a-c) Isosurface plots for SO densities of the reactant (a), transition state (b) and product (c) for the reaction TN19 HCN $\rightarrow$ HNC. All isosurface values are 0.001/Bohr^2. Color: C (brown), H (pink), N (blue), unpolarized PO (purple). The SO labels are the same as in Table S7. The orbital indices shown here were automatically generated by the FLOSIC code for the TS and manually mapped to the corresponding orbitals for R and P.

Table S7: (i) SIC energies U_i^{SIC} (Ha) computed in PZSIC and LSIC for the participant orbitals (POs) of the reactant (R), transition state (TS), and product (P) for the reaction TN19 HCN → HNC. Columns 2, 3, and 4 give the PZSIC energies. Columns 5 and 6 give the difference, $\Delta U_i^{\mathrm{SIC}}$ (TS−R) and $\Delta U_i^{\mathrm{SIC}}$ (TS−P). The labels in column 1 are the same as those used in Fig. S3. The sum of the differences is shown in the last row. (ii) Same as in (i), but for the spectator orbitals (SOs). The labels in column 1 are the same as those used in Fig. S4.

(i) PO SIC energies

PO	U_i^{SIC}(R)	U_i^{SIC}(TS)	U_i^{SIC} (P)	$\Delta U_i^{\mathrm{SIC}}$ (TS−R)	$\Delta U_i^{\mathrm{SIC}}$(TS−P)
5	-0.0112	-0.0099	-0.0128	0.0013	0.0029
14	-0.0112	-0.0099	-0.0128	0.0013	0.0029
4	-0.0254	-0.0142	-0.0182	0.0112	0.0040
10	-0.0254	-0.0142	-0.0182	0.0112	0.0040
7	-0.0239	-0.0241	-0.0278	-0.0002	0.0037
13	-0.0239	-0.0241	-0.0278	-0.0002	0.0037
$\Delta E_{\mathrm{PO}}^{\mathrm{SIC}}$ (TS-R)				0.0246	
$\Delta E_{\mathrm{PO}}^{\mathrm{SIC}}$ (TS-P)					0.0212

(ii) SO SIC energies

SO	U_i^{SIC}(R)	U_i^{SIC}(TS)	U_i^{SIC} (P)	$\Delta U_i^{\mathrm{SIC}}$ (TS−R)	$\Delta U_i^{\mathrm{SIC}}$(TS−P)
1	-0.2081	-0.2085	-0.2089	-0.0004	0.0004
6	-0.2515	-0.2517	-0.2507	-0.0002	-0.0010
8	-0.2081	-0.2085	-0.2089	-0.0004	0.0004
9	-0.2515	-0.2517	-0.2507	-0.0002	-0.0010
2	-0.0112	-0.0158	-0.0128	-0.0046	-0.0030
3	-0.0112	-0.0159	-0.0129	-0.0047	-0.0030
11	-0.0112	-0.0159	-0.0128	-0.0047	-0.0031
12	-0.0112	-0.0158	-0.0129	-0.0046	-0.0029
$\Delta E_{\mathrm{SO}}^{\mathrm{SIC}}$ (TS-R)				-0.0198	
$\Delta E_{\mathrm{SO}}^{\mathrm{SIC}}$ (TS-P)					-0.0132

Table S8: The set of BH76 reactions that follow the hypothesis for LSIC calculations. Energies are reported in Ha. Frac (PO) is calculated as the ratio of $\Delta E_{\text{PO}}^{\text{SIC}}/\Delta E_{\text{Total}}^{\text{SIC}}$. Similarly, Frac (SO) is calculated as the ratio of $\Delta E_{\text{SO}}^{\text{SIC}}/\Delta E_{\text{Total}}^{\text{SIC}}$.

Label	Reaction	$\Delta E_{\text{PO}}^{\text{SIC}}$	Frac(PO)	$\Delta E_{\text{SO}}^{\text{SIC}}$	Frac(SO)	$\Delta E_{\text{Total}}^{\text{SIC}}$
TN3(F)	$H + ClH \rightarrow HCl + H$	0.0172	0.76	0.0053	0.24	0.0225
TN4(F)	$H + FCH_3 \rightarrow HF + CH_3$	0.0157	0.77	0.0047	0.23	0.0204
TN5(R)	$H + F_2 \rightarrow HF + F$	0.0434	0.85	0.0074	0.15	0.0508
TN6(R)	$CH_3 + FCl \rightarrow CH_3F + Cl$	0.0206	0.83	0.0042	0.17	0.0248
TN8(F)	$F^- ... CH_3F \rightarrow FCH_3 ... F^-$	0.0110	1.08	-0.0008	-0.08	0.0102
TN9(F)	$Cl^- + CH_3Cl \rightarrow ClCH_3 + Cl^-$	0.0140	0.70	0.0059	0.30	0.0199
TN10(F)	$Cl^- ... CH_3Cl \rightarrow ClCH_3 ... Cl^-$	0.0092	1.11	-0.0009	-0.11	0.0083
TN13(R)	$OH^- + CH_3F \rightarrow HOCH_3 + F^-$	0.0218	0.82	0.0048	0.18	0.0266
TN14(F)	$OH^- ... CH_3F \rightarrow HOCH_3 ... F^-$	0.0135	1.18	-0.0021	-0.18	0.0114
TN15(F)	$H + N_2 \rightarrow HN_2$	0.0193	1.01	0.0000	-0.01	0.0192
TN15(R)	$H + N_2 \rightarrow HN_2$	0.0177	1.16	-0.0025	-0.16	0.0152
TN16(F)	$H + CO \rightarrow HCO$	0.0111	0.81	0.0026	0.19	0.0137
TN16(R)	$H + CO \rightarrow HCO$	0.0081	1.08	-0.0006	-0.08	0.0075
TN17(F)	$H + C_2H_4 \rightarrow CH_3CH_2$	0.0140	0.90	0.0015	0.10	0.0155
T1(F)	$H + HCl \rightarrow H_2 + Cl$	0.0119	1.11	-0.0012	-0.11	0.0107
T3(F)	$CH_3 + H_2 \rightarrow CH_4 + H$	0.0163	0.70	0.0071	0.30	0.0234
T4(R)	$OH + CH_4 \rightarrow H_2O + CH_3$	0.0162	0.81	0.0038	0.19	0.0200
T5(F)	$H + H_2 \rightarrow H_2 + H$	0.0163	1.00	0.0000	0.00	0.0163
T6(R)	$OH + NH_3 \rightarrow H_2O + NH_2$	0.0248	0.71	0.0101	0.29	0.0349
T7(R)	$HCl + CH_3 \rightarrow CH_4 + Cl$	0.0203	0.74	0.0073	0.26	0.0276
T8(R)	$OH + C_2H_6 \rightarrow H_2O + C_2H_5$	0.0136	0.87	0.0021	0.13	0.0157
T10(R)	$O + CH_4 \rightarrow OH + CH_3$	0.0187	0.75	0.0064	0.25	0.0251
T11(F)	$H + PH_3 \rightarrow H_2 + PH_2$	0.0114	0.88	0.0016	0.12	0.0130
T11(R)	$H + PH_3 \rightarrow H_2 + PH_2$	0.0202	0.75	0.0069	0.25	0.0271
T13(F)	$H + H_2S \rightarrow H_2 + HS$	0.0117	0.95	0.0006	0.05	0.0123
T13(R)	$H + H_2S \rightarrow H_2 + HS$	0.0201	0.70	0.0085	0.30	0.0286
T14(R)	$O + HCl \rightarrow OH + Cl$	0.0258	0.75	0.0084	0.25	0.0342
T17(R)	$NH_2 + C_2H_6 \rightarrow NH_3 + C_2H_5$	0.0143	0.71	0.0058	0.29	0.0201
T19(F)	$s{-}transcis{-}C_5H_8 \rightarrow s{-}transcis{-}C_5H_8$	0.0489	1.00	0.0002	0.00	0.0491
T1(R)	$H + HCl \rightarrow H_2 + Cl$	0.0201	0.69	0.0089	0.31	0.0290
T12(F)	$H + HO \rightarrow H_2 + O$	0.0193	1.24	-0.0037	-0.24	0.0156
T15(R)	$CH_3 + NH_2 \rightarrow CH_4 + NH$	0.0210	0.67	0.0103	0.33	0.0313
T18(R)	$NH_2 + CH_4 \rightarrow NH_3 + CH_3$	0.0163	0.67	0.0081	0.33	0.0244
TN2(F)	$H + FH \rightarrow HF + H$	0.0207	0.69	0.0094	0.31	0.0301

Table S9: The set of BH76 reactions that fail the hypothesis for LSIC calculations. Energies are reported in Ha. Frac (PO) is calculated as the ratio of $\Delta E_{\mathrm{PO}}^{\mathrm{SIC}}/\Delta E_{\mathrm{Total}}^{\mathrm{SIC}}$. Similarly, Frac (SO) is calculated as the ratio of $\Delta E_{\mathrm{SO}}^{\mathrm{SIC}}/\Delta E_{\mathrm{Total}}^{\mathrm{SIC}}$.

Label	Reaction	$\Delta E_{\mathrm{PO}}^{\mathrm{SIC}}$	Frac(PO)	$\Delta E_{\mathrm{SO}}^{\mathrm{SIC}}$	Frac(SO)	$\Delta E_{\mathrm{Total}}^{\mathrm{SIC}}$
T2(F)	$OH + H_2 \rightarrow H_2O + H$	0.0225	0.65	0.0121	0.35	0.0346
T3(R)	$CH_3 + H_2 \rightarrow CH_4 + H$	0.0168	1.37	-0.0045	-0.37	0.0123
T4(F)	$OH + CH_4 \rightarrow H_2O + CH_3$	0.0222	0.63	0.0131	0.37	0.0353
T6(F)	$OH + NH_3 \rightarrow H_2O + NH_2$	0.0254	0.63	0.0152	0.37	0.0406
T7(F)	$HCl + CH_3 \rightarrow CH_4 + Cl$	0.0116	0.57	0.0088	0.43	0.0204
T8(F)	$OH + C_2H_6 \rightarrow H_2O + C_2H_5$	0.0213	0.60	0.0144	0.40	0.0357
T9(F)	$F + H_2 \rightarrow HF + H$	0.0160	0.53	0.0144	0.47	0.0304
T10(F)	$O + CH_4 \rightarrow OH + CH_3$	0.0233	0.63	0.0136	0.37	0.0369
T12(R)	$H + HO \rightarrow H_2 + O$	0.0234	0.61	0.0151	0.39	0.0385
T14(F)	$O + HCl \rightarrow OH + Cl$	0.0217	0.56	0.0171	0.44	0.0388
T15(F)	$CH_3 + NH_2 \rightarrow CH_4 + NH$	0.0167	0.66	0.0085	0.34	0.0252
T16(F)	$C_2H_5 + NH_2 \rightarrow C_2H_6 + NH$	0.0150	0.66	0.0076	0.34	0.0226
T16(R)	$C_2H_5 + NH_2 \rightarrow C_2H_6 + NH$	0.0210	0.63	0.0124	0.37	0.0334
T17(F)	$NH_2 + C_2H_6 \rightarrow NH_3 + C_2H_5$	0.0214	0.62	0.0130	0.38	0.0344
T18(F)	$NH_2 + CH_4 \rightarrow NH_3 + CH_3$	0.0217	0.64	0.0123	0.48	0.0340
TN1(R)	$H + N_2O \rightarrow OH + N_2$	0.0509	0.62	0.0307	0.38	0.0816
TN4(R)	$H + FCH_3 \rightarrow HF + CH_3$	0.0242	0.56	0.0191	0.44	0.0433
TN5(F)	$H + F_2 \rightarrow HF + F$	0.0036	0.30	0.0083	0.70	0.0119
TN7(F)	$F^- + CH_3F \rightarrow FCH_3 + F^-$	0.0170	0.65	0.0092	0.35	0.0262
TN12(F)	$F^- ... CH_3Cl \rightarrow FCH_3 ... Cl^-$	0.0106	1.54	-0.0037	-0.54	0.0069
TN18(F)	$CH_3 + C_2H_4 \rightarrow CH_3CH_2CH_2$	0.0135	0.52	0.0125	0.48	0.0260
TN1(F)	$H + N_2O \rightarrow OH + N_2$	0.0195	3.42	-0.0138	-2.42	0.0057
TN6(F)	$CH_3 + FCl \rightarrow CH_3F + Cl$	0.0030	0.19	0.0127	0.81	0.0157
TN11(R)	$F^- + CH_3Cl \rightarrow FCH_3 + Cl^-$	0.0038	0.20	0.0151	0.80	0.0189
TN12(R)	$F^- ... CH_3Cl \rightarrow FCH_3 ... Cl^-$	-0.0022	0.05	-0.0396	0.95	-0.0418
TN14(R)	$OH^- ... CH_3F \rightarrow HOCH_3 ... F^-$	0.0122	7.62	-0.0106	-6.62	0.0016
TN17(R)	$H + C_2H_4 \rightarrow CH_3CH_2$	0.0084	2.15	-0.0045	-1.15	0.0039
TN18(R)	$CH_3 + C_2H_4 \rightarrow CH_3CH_2CH_2$	0.0058	-0.61	-0.0153	1.61	-0.0095
TN19(F)	$HCN \rightarrow HNC$	0.0168	3.17	-0.0115	-2.17	0.0053
TN19(R)	$HCN \rightarrow HNC$	0.0134	1.86	-0.0062	-0.86	0.0072
T2(R)	$OH + H_2 \rightarrow H_2O + H$	0.0170	2.07	-0.0088	-1.07	0.0082
T9(R)	$F + H_2 \rightarrow HF + H$	0.0106	-7.07	-0.0121	8.07	-0.0015

Table S10: BH76 reactions that follow the hypothesis for PZSIC@LDA for the subset of reactions tested. The reactions studied are: T1,T3,T4,T5,T6,T7,T9,T10,T12,T15, TN2,TN4,TN7,TN8,TN9,TN15,TN16,TN19. $\Delta E^{\mathrm{SIC}}_{\mathrm{PO}}$, $\Delta E^{\mathrm{SIC}}_{\mathrm{SO}}$ and $\Delta E^{\mathrm{SIC}}_{\mathrm{Total}}$ are reported in Ha. Frac (PO) is calculated as the ratio of $\Delta E^{\mathrm{SIC}}_{\mathrm{PO}}/\Delta E^{\mathrm{SIC}}_{\mathrm{Total}}$. Similarly, Frac (SO) is calculated as the ratio of $\Delta E^{\mathrm{SIC}}_{\mathrm{SO}}/\Delta E^{\mathrm{SIC}}_{\mathrm{Total}}$.

Label	Reaction	$\Delta E^{\mathrm{SIC}}_{\mathrm{PO}}$	Frac(PO)	$\Delta E^{\mathrm{SIC}}_{\mathrm{SO}}$	Frac(SO)	$\Delta E^{\mathrm{SIC}}_{\mathrm{Total}}$
T1(F)	$H + HCl \rightarrow H_2 + Cl$	0.0157	1.19	-0.0025	-0.19	0.0132
T4(R)	$OH + CH_4 \rightarrow H_2O + CH_3$	0.0173	1.10	-0.0016	-0.10	0.0157
T4(F)	$OH + CH_4 \rightarrow H_2O + CH_3$	0.0298	0.72	0.0114	0.28	0.0413
T5(F)	$H + H_2 \rightarrow H_2 + H$	0.0152	1.00	0.0000	0.00	0.0152
T6(F)	$OH + NH_3 \rightarrow H_2O + NH_2$	0.0473	0.88	0.0064	0.12	0.0536
T6(R)	$OH + NH_3 \rightarrow H_2O + NH_2$	0.0428	1.00	0.0000	0.00	0.0429
T7(F)	$HCl + CH_3 \rightarrow CH_4 + Cl$	0.0094	0.69	0.0043	0.31	0.0136
T7(R)	$HCl + CH_3 \rightarrow CH_4 + Cl$	0.0220	0.78	0.0061	0.22	0.0281
T9(F)	$F + H_2 \rightarrow HF + H$	0.0346	0.69	0.0155	0.31	0.0500
T10(F)	$O + CH_4 \rightarrow OH + CH_3$	0.0317	0.74	0.0111	0.26	0.0428
T10(R)	$O + CH_4 \rightarrow OH + CH_3$	0.0174	0.88	0.0025	0.12	0.0199
T12(F)	$H + HO \rightarrow H_2 + O$	0.0266	1.16	-0.0036	-0.16	0.0229
T15(R)	$CH_3 + NH_2 \rightarrow CH_4 + NH$	0.0268	0.83	0.0056	0.17	0.0325
T3(R)	$CH_3 + H_2 \rightarrow CH_4 + H$	0.0214	1.25	-0.0042	-0.25	0.0172
T15(F)	$CH_3 + NH_2 \rightarrow CH_4 + NH$	0.0153	0.71	0.0064	0.29	0.0217
TN2(F)	$H + FH \rightarrow HF + H$	0.0714	1.03	-0.0022	-0.03	0.0692
TN4(F)	$H + FCH_3 \rightarrow HF + CH_3$	0.0668	0.99	0.0008	0.01	0.0676
TN4(R)	$H + FCH_3 \rightarrow HF + CH_3$	0.0594	1.05	-0.0026	-0.05	0.0568
TN7(F)	$F^- + CH_3F \rightarrow FCH_3 + F^-$	0.0336	1.21	-0.0058	-0.21	0.0278
TN9(F)	$Cl^- + CH_3Cl \rightarrow ClCH_3 + Cl^-$	0.0286	1.08	-0.0022	-0.08	0.0264
TN15(R)	$H + N_2 \rightarrow HN_2$	0.0388	1.22	-0.0071	-0.22	0.0318
TN16(F)	$H + CO \rightarrow HCO$	0.0125	0.79	0.0032	0.21	0.0157
TN16(R)	$H + CO \rightarrow HCO$	0.0191	0.97	0.0006	0.03	0.0197

Table S11: BH76 reactions that fail the hypothesis for PZSIC@LDA for the subset of reactions tested. The reactions studied are identified in Table S10. $\Delta E_{\mathrm{PO}}^{\mathrm{SIC}}$, $\Delta E_{\mathrm{SO}}^{\mathrm{SIC}}$ and $\Delta E_{\mathrm{Total}}^{\mathrm{SIC}}$ are reported in Ha. Frac (PO) is calculated as the ratio of $\Delta E_{\mathrm{PO}}^{\mathrm{SIC}}/\Delta E_{\mathrm{Total}}^{\mathrm{SIC}}$. Similarly, Frac (SO) is calculated as the ratio of $\Delta E_{\mathrm{SO}}^{\mathrm{SIC}}/\Delta E_{\mathrm{Total}}^{\mathrm{SIC}}$.

Label	Reaction	$\Delta E_{\mathrm{PO}}^{\mathrm{SIC}}$	Frac(PO)	$\Delta E_{\mathrm{SO}}^{\mathrm{SIC}}$	Frac(SO)	$\Delta E_{\mathrm{Total}}^{\mathrm{SIC}}$
T1(R)	$H + HCl \rightarrow H_2 + Cl$	0.0141	0.64	0.0079	0.36	0.0220
T2(F)	$OH + H_2 \rightarrow H_2O + H$	0.0251	0.66	0.0128	0.34	0.0379
T2(R)	$OH + H_2 \rightarrow H_2O + H$	0.0268	1.49	-0.0088	-0.49	0.0180
T3(F)	$CH_3 + H_2 \rightarrow CH_4 + H$	0.0072	0.63	0.0043	0.37	0.0115
T9(R)	$F + H_2 \rightarrow HF + H$	0.0316	1.88	-0.0148	-0.88	0.0168
T12(R)	$H + HO \rightarrow H_2 + O$	0.0266	0.66	0.0135	0.34	0.0401
TN8(F)	$F^- \ldots CH_3F \rightarrow FCH_3 \ldots F^-$	0.0294	1.37	-0.0079	-0.37	0.0214
TN15(F)	$H + N_2 \rightarrow HN_2$	0.0415	1.39	-0.0116	-0.39	0.0299
TN19(F)	$HCN \rightarrow HNC$	0.0312	2.01	-0.0156	-1.01	0.0155
TN19(R)	$HCN \rightarrow HNC$	0.0281	1.52	-0.0097	-0.52	0.0184

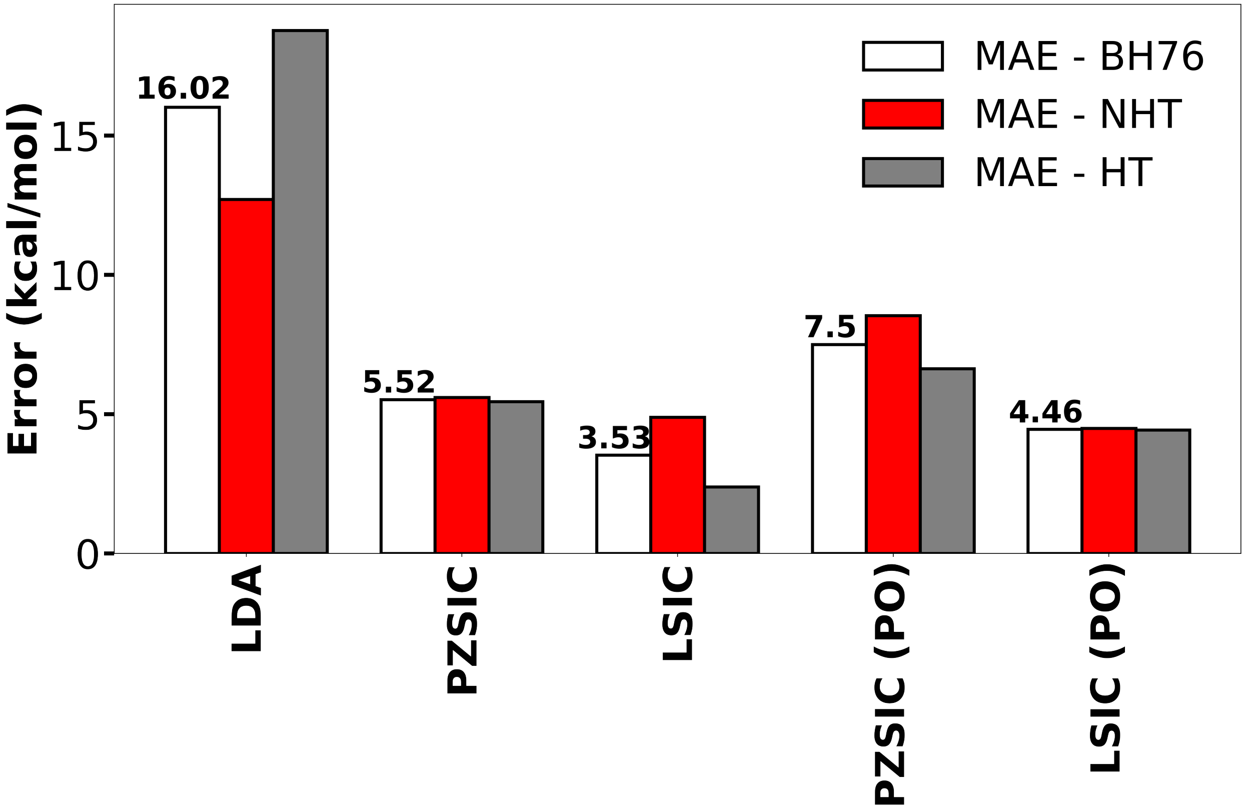

Figure S5: Mean absolute error (MAE) with respect to the reference barrier heights for LDA, PZSIC, LSIC, PZSIC (PO), and LSIC (PO) for the 66 distinct reactions in BH76 database and for NHT and HT reactions.

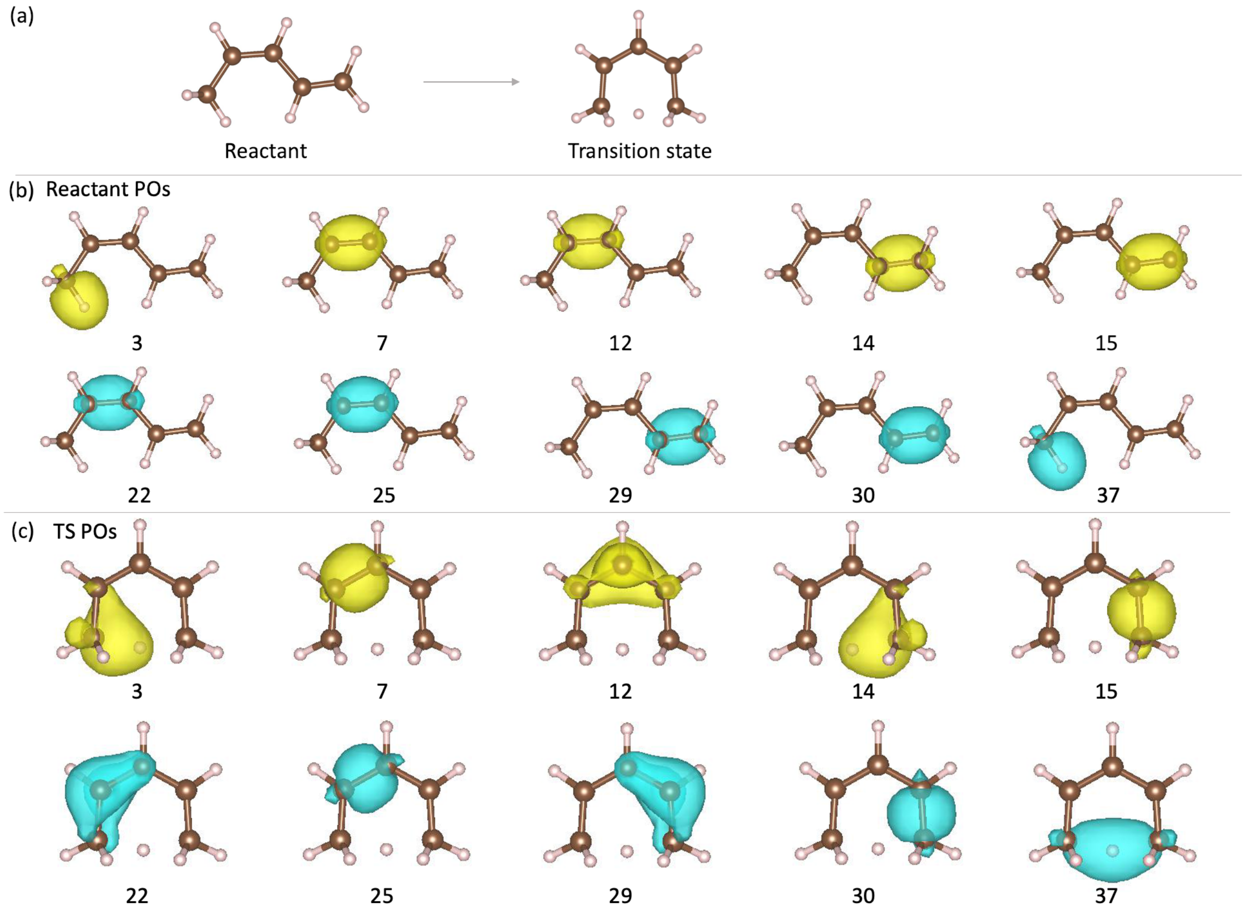

Figure S6: (a) The reaction scheme for T19 (s−transcis−C_5H_8→s−transcis−C_5H_8). (b-c) Isosurface plots of PO densities for the reactants (b) and the transition state (c). All isosurface values are 0.001/$Bohr^2$. Color: H (pink), C (brown), up-spin PO (yellow), and down-spin PO (cyan). The labels of the PO are the same as in Table S12. The orbital indices shown here were automatically generated by the FLOSIC code for the TS and manually mapped to the corresponding orbitals for R and P.

Table S12: (i) SIC energies U_i^{SIC} (Ha) computed from PZSIC and LSIC for the POs of the (R) and (TS) for T19. PZSIC: columns 2 and 3. LSIC: columns 5 and 6. Columns 4 and 7 give the difference, ΔU_i^{SIC} (TS-R), for PZSIC and LSIC respectively. The sum of the differences is shown in the last row. The labels in column 1 are the same as those used in Fig. S6. (ii) Same as in (i), but for the SOs.

(i) PO SIC energies

PO	U_i^{SIC}(R)	U_i^{SIC}(TS)	ΔU_i^{SIC} (TS−R)	U_i^{LSIC} (R)	U_i^{LSIC} (TS)	ΔU_i^{LSIC}(TS−R)
3	-0.0223	-0.0059	0.0164	-0.0159	-0.0065	0.0094
7	-0.0117	-0.0241	-0.0124	-0.0102	-0.0124	-0.0022
12	-0.0117	0.0066	0.0183	-0.0102	-0.0016	0.0086
14	-0.0115	-0.0060	0.0055	-0.0108	-0.0065	0.0043
15	-0.0115	-0.0224	-0.0109	-0.0108	-0.0117	-0.0009
22	-0.0117	0.0093	0.0210	-0.0102	-0.0003	0.0099
25	-0.0117	-0.0245	-0.0128	-0.0102	-0.0117	-0.0015
29	-0.0115	0.0089	0.0204	-0.0108	-0.0006	0.0102
30	-0.0115	-0.0239	-0.0124	-0.0108	-0.0114	-0.0006
37	-0.0223	-0.0065	0.0158	-0.0159	-0.0042	0.0117
$\Delta E_{\text{PO}}^{\text{SIC}}(\text{TS} - \text{P})$			0.0489			0.0489

(ii) SO SIC energies

SO	U_i^{SIC}(R)	U_i^{SIC}(TS)	ΔU_i^{SIC} (TS−R)	U_i^{LSIC} (R)	U_i^{LSIC} (TS)	ΔU_i^{LSIC}(TS−R)
1	-0.2072	-0.2075	-0.0003	-0.1545	-0.1525	0.0020
2	-0.0210	-0.0223	-0.0013	-0.0111	-0.0117	-0.0006
4	-0.0217	-0.0227	-0.0010	-0.0164	-0.0170	-0.0006
5	-0.0217	-0.0217	0.0000	-0.0164	-0.0151	0.0013
6	-0.2076	-0.2074	0.0002	-0.155	-0.1575	-0.0025
8	-0.0235	-0.0238	-0.0003	-0.0171	-0.0176	-0.0005
9	-0.2075	-0.2074	0.0000	-0.1547	-0.1530	0.0017
10	-0.0231	-0.0241	-0.0010	-0.0115	-0.0124	-0.0009
11	-0.0234	-0.0236	-0.0002	-0.0167	-0.0173	-0.0006
13	-0.2076	-0.2074	0.0002	-0.1548	-0.1575	-0.0027
16	-0.0234	-0.0238	-0.0004	-0.0164	-0.0176	-0.0012
17	-0.2077	-0.2075	0.0002	-0.1548	-0.1525	0.0023
18	-0.0235	-0.0227	0.0008	-0.0178	-0.0170	0.0008
19	-0.0237	-0.0217	0.0020	-0.0182	-0.0151	0.0031
20	-0.2072	-0.2072	0.0000	-0.1545	-0.1576	-0.0031
21	-0.2076	-0.2072	0.0004	-0.1550	-0.1521	0.0029
23	-0.0210	-0.0241	-0.0031	-0.0111	-0.0115	-0.0004
24	-0.2075	-0.2072	0.0003	-0.1547	-0.1562	-0.0015
26	-0.2076	-0.2075	0.0000	-0.1548	-0.1523	0.0025
27	-0.0231	-0.0244	-0.0013	-0.0115	-0.0116	0.0000
28	-0.2077	-0.2072	0.0005	-0.1548	-0.1576	-0.0028
31	-0.0235	-0.0236	0.0000	-0.0178	-0.0177	0.0000
32	-0.0237	-0.0229	0.0008	-0.0182	-0.0155	0.0027
33	-0.0235	-0.0234	0.0000	-0.0164	-0.0169	-0.0005
34	-0.0234	-0.0241	-0.0007	-0.0167	-0.0175	-0.0008
35	-0.0235	-0.0238	-0.0003	-0.0171	-0.0171	0.0000
36	-0.0217	-0.0234	-0.0017	-0.0164	-0.0175	-0.0011
38	-0.0217	-0.0231	-0.0014	-0.0164	-0.0157	0.0007
$\Delta E_{\text{SO}}^{\text{SIC}}(\text{TS} - \text{P})$			-0.0074			0.0002

Table S13: Difference between the LSIC and PZSIC SIC energies (Ha) for the POs and SOs for the non-hydrogen transfer reactions.

Label	Reactions	ΔE_{PO}^{SIC}	ΔE_{SO}^{SIC}	ΔE_{PO}^{SIC}	ΔE_{SO}^{SIC}	diff ΔE_{SO}^{SIC}	diff ΔE_{PO}^{SIC}
		PZSIC	PZSIC	LSIC	LSIC	LSIC−PZSIC	LSIC−PZSIC
TN1(F)	$H + N_2O \rightarrow OH + N_2$	0.0218	-0.0431	0.0195	-0.0138	0.0293	-0.0023
TN1 (R)	$H + N_2O \rightarrow OH + N_2$	0.0399	0.0039	0.0509	0.0307	0.0268	0.0110
TN2(F)	$H + FH \rightarrow HF + H$	0.0255	-0.0005	0.0207	0.0094	0.0099	-0.0048
TN3(F)	$H + ClH \rightarrow HCl + H$	0.0172	0.0020	0.0172	0.0053	0.0033	0.0000
TN4(F)	$H + FCH_3 \rightarrow HF + CH_3$	0.0399	-0.0043	0.0157	0.0047	0.0090	-0.0242
TN4(R)	$H + FCH_3 \rightarrow HF + CH_3$	0.0350	-0.0101	0.0242	0.0191	0.0292	-0.0108
TN5(F)	$H + F_2 \rightarrow HF + F$	-0.0079	0.0105	0.0036	0.0083	-0.0022	0.0115
TN5(R)	$H + F_2 \rightarrow HF + F$	0.0563	-0.0264	0.0434	0.0074	0.0338	-0.0129
TN6(F)	$CH_3 + FCl \rightarrow CH_3F + Cl$	0.0176	-0.0002	0.0030	0.0127	0.0129	-0.0146
TN6(R)	$CH_3 + FCl \rightarrow CH_3F + Cl$	0.0433	-0.0111	0.0206	0.0042	0.0153	-0.0227
TN7(F)	$F^- + CH_3F \rightarrow FCH_3 + F^-$	0.0207	-0.0016	0.0170	0.0092	0.0108	-0.0037
TN8(F)	$F^- ... CH_3F \rightarrow FCH_3 ... F^-$	0.0192	-0.0032	0.0110	-0.0008	0.0024	-0.0082
TN9(F)	$Cl^- + CH_3Cl \rightarrow ClCH_3 + Cl^-$	0.0202	-0.0011	0.0140	0.0059	0.0070	-0.0062
TN10(F)	$Cl^- ... CH_3Cl \rightarrow ClCH_3 ... Cl^-$	0.0180	-0.0053	0.0092	-0.0009	0.0044	-0.0088
TN11(R)	$F^- + CH_3Cl \rightarrow FCH_3 + Cl^-$	0.0068	0.0147	0.0038	0.0151	0.0004	-0.003
TN12(F)	$F^- ... CH_3Cl \rightarrow FCH_3 ... Cl^-$	0.0174	-0.0076	0.0106	-0.0037	0.0039	-0.0068
TN12(R)	$F^- ... CH_3Cl \rightarrow FCH_3 ... Cl^-$	0.0058	0.0118	0.0000	0.0087	-0.0031	-0.0058
TN13(R)	$OH^- + CH_3F \rightarrow HOCH_3 + F^-$	0.0293	-0.0073	0.0218	0.0048	0.0121	-0.0075
TN14(F)	$OH^- ... CH_3F \rightarrow HOCH_3 ... F^-$	0.0174	0.0002	0.0135	-0.0021	-0.0023	-0.0039
TN14(R)	$OH^- ... CH_3F \rightarrow HOCH_3 ... F^-$	0.0280	-0.0113	0.0122	-0.0106	0.0007	-0.0158
TN15(F)	$H + N_2 \rightarrow HN_2$	0.0212	-0.0138	0.0193	0.0000	0.0137	-0.0019
TN15(R)	$H + N_2 \rightarrow HN_2$	0.0301	-0.0057	0.0177	-0.0025	0.0032	-0.0124
TN16(F)	$H + CO \rightarrow HCO$	0.0052	0.0015	0.0111	0.0026	0.0011	0.0059
TN16(R)	$H + CO \rightarrow HCO$	0.0124	0.0025	0.0081	-0.0006	-0.0031	-0.0043
TN17(F)	$H + C_2H_4 \rightarrow CH_3CH_2$	0.0058	-0.0005	0.014	0.0015	0.0020	0.0082
TN17(R)	$H + C_2H_4 \rightarrow CH_3CH_2$	0.0218	-0.0115	0.0084	-0.0045	0.0070	-0.0134
TN18(F)	$CH_3 + C_2H_4 \rightarrow CH_3CH_2CH_2$	0.0019	0.0013	0.0135	0.0125	0.0112	0.0116
TN18(R)	$CH_3 + C_2H_4 \rightarrow CH_3CH_2CH_2$	0.0286	-0.0151	0.0058	-0.0153	-0.0002	-0.0228
TN19(F)	$HCN \rightarrow HNC$	0.0246	-0.0198	0.0168	-0.0115	0.0083	-0.0078
TN19(R)	$HCN \rightarrow HNC$	0.0212	-0.0132	0.0134	-0.0062	0.0070	-0.0078
Absolute average						0.009	0.009
Signed average						0.008	-0.006

Table S14: Difference between LSIC and PZSIC SIC energies (Ha) for the POs and SOs for the hydrogen transfer reactions.

Label	Reactions	$\Delta E_{\mathrm{PO}}^{\mathrm{SIC}}$	$\Delta E_{\mathrm{SO}}^{\mathrm{SIC}}$	$\Delta E_{\mathrm{PO}}^{\mathrm{SIC}}$	$\Delta E_{\mathrm{SO}}^{\mathrm{SIC}}$	diff $\Delta E_{\mathrm{SO}}^{\mathrm{SIC}}$	diff $\Delta E_{\mathrm{PO}}^{\mathrm{SIC}}$
		PZSIC	PZSIC	LSIC	LSIC	LSIC−PZSIC	LSIC−PZSIC
T1(F)	$H + HCl \rightarrow H_2 + Cl$	0.0107	-0.0025	0.0119	-0.0012	0.0013	0.0012
T1(R)	$H + HCl \rightarrow H_2 + Cl$	0.0107	0.0054	0.0201	0.0089	0.0035	0.0094
T2(F)	$OH + H_2 \rightarrow H_2O + H$	0.0162	0.0058	0.0225	0.0121	0.0063	0.0063
T2(R)	$OH + H_2 \rightarrow H_2O + H$	0.0157	-0.0122	0.0170	-0.0088	0.0034	0.0013
T3(F)	$CH_3 + H_2 \rightarrow CH_4 + H$	0.0035	0.0014	0.0163	0.0071	0.0057	0.0128
T3(R)	$CH_3 + H_2 \rightarrow CH_4 + H$	0.0174	-0.004	0.0168	-0.0045	-0.0005	-0.0006
T4(F)	$OH + CH_4 \rightarrow H_2O + CH_3$	0.0219	0.0055	0.0222	0.0131	0.0076	0.0003
T4(R)	$OH + CH_4 \rightarrow H_2O + CH_3$	0.0075	-0.0071	0.0162	0.0038	0.0109	0.0087
T5(F)	$H + H_2 \rightarrow H_2 + H$	0.0115	0.0000	0.0163	0.0000	0.0000	0.0048
T6(F)	$OH + NH_3 \rightarrow H_2O + NH_2$	0.0301	0.0045	0.0254	0.0152	0.0107	-0.0047
T6(R)	$OH + NH_3 \rightarrow H_2O + NH_2$	0.0264	-0.0026	0.0248	0.0101	0.0127	-0.0016
T7(F)	$HCl + CH_3 \rightarrow CH_4 + Cl$	0.0056	0.0011	0.0116	0.0088	0.0077	0.0060
T7(R)	$HCl + CH_3 \rightarrow CH_4 + Cl$	0.0195	0.0036	0.0203	0.0073	0.0037	0.0008
T8(F)	$OH + C_2H_6 \rightarrow H_2O + C_2H_5$	0.0203	0.0062	0.0213	0.0144	0.0082	0.0010
T8(R)	$OH + C_2H_6 \rightarrow H_2O + C_2H_5$	0.0024	-0.0070	0.0136	0.0021	0.0091	0.0112
T9(F)	$F + H_2 \rightarrow HF + H$	0.0132	0.0064	0.016	0.0144	0.0080	0.0028
T9(R)	$F + H_2 \rightarrow HF + H$	0.0090	-0.0195	0.0106	-0.0121	0.0074	0.0016
T10(F)	$O + CH_4 \rightarrow OH + CH_3$	0.0257	0.0059	0.0233	0.0136	0.0077	-0.0024
T10(R)	$O + CH_4 \rightarrow OH + CH_3$	0.0105	-0.0021	0.0187	0.0064	0.0085	0.0082
T11(F)	$H + PH_3 \rightarrow H_2 + PH_2$	0.009	0.0007	0.0114	0.0016	0.0009	0.0024
T11(R)	$H + PH_3 \rightarrow H_2 + PH_2$	0.0062	0.0024	0.0202	0.0069	0.0045	0.0140
T12(F)	$H + HO \rightarrow H_2 + O$	0.0197	-0.0058	0.0193	-0.0037	0.0021	-0.0004
T12(R)	$H + HO \rightarrow H_2 + O$	0.021	0.0076	0.0234	0.0151	0.0075	0.0024
T13(F)	$H + H_2S \rightarrow H_2 + HS$	0.0104	0.0002	0.0117	0.0006	0.0004	0.0013
T13(R)	$H + H_2S \rightarrow H_2 + HS$	0.0081	0.0048	0.0201	0.0085	0.0036	0.0120
T14(F)	$O + HCl \rightarrow OH + Cl$	0.0268	0.0052	0.0217	0.0171	0.0119	-0.0051
T14(R)	$O + HCl \rightarrow OH + Cl$	0.0255	-0.0003	0.0258	0.0084	0.0087	0.0003
T15(F)	$CH_3 + NH_2 \rightarrow CH_4 + NH$	0.0089	0.0025	0.0167	0.0085	0.006	0.0078
T15(R)	$CH_3 + NH_2 \rightarrow CH_4 + NH$	0.0205	0.0029	0.0210	0.0103	0.0074	0.0005
T16(F)	$C_2H_5 + NH_2 \rightarrow C_2H_6 + NH$	0.0058	0.0029	0.0150	0.0076	0.0047	0.0092
T16(R)	$C_2H_5 + NH_2 \rightarrow C_2H_6 + NH$	0.0206	0.0042	0.0210	0.0124	0.0082	0.0004
T17(F)	$NH_2 + C_2H_6 \rightarrow NH_3 + C_2H_5$	0.0202	0.0047	0.0214	0.013	0.0083	0.0012
T17(R)	$NH_2 + C_2H_6 \rightarrow NH_3 + C_2H_5$	0.006	-0.0014	0.0143	0.0058	0.0072	0.0083
T18(F)	$NH_2 + CH_4 \rightarrow NH_3 + CH_3$	0.0203	0.0062	0.0217	0.0123	0.0061	0.0014
T18(R)	$NH_2 + CH_4 \rightarrow NH_3 + CH_3$	0.0093	0.0010	0.0163	0.0081	0.0071	0.0070
T19(F)	$s{-}transcis{-}C_5H_8 \rightarrow s{-}transcis{-}C_5H_8$	0.0489	-0.0074	0.0489	0.0002	0.0076	0.0000
Absolute average						0.006	0.004
Signed average						0.006	0.004
